# Ochraceopetalin, a Mixed-Biogenetic Salt of Polyketide and Amino Acid Origins from a Marine-Derived *Aspergillus ochraceopetaliformis* Fungus

**DOI:** 10.3390/md19080413

**Published:** 2021-07-26

**Authors:** Sung Chul Park, Jung-Ho Lee, Ji-Yeon Hwang, Oh-Seok Kwon, Lijuan Liao, Dong-Chan Oh, Ki-Bong Oh, Jongheon Shin

**Affiliations:** 1 Natural Products Research Institute, College of Pharmacy, Seoul National University, San 56-1, Sillim, Gwanak, Seoul 151-742, Korea; sungchulpark@snu.ac.kr (S.C.P.); anilove@snu.ac.kr (J.-H.L.); yahyah7@snu.ac.kr (J.-Y.H.); ideally225@snu.ac.kr (O.-S.K.); lxycxf@snu.ac.kr (L.L.); dongchanoh@snu.ac.kr (D.-C.O.); 2 College of Life Sciences, Fujian Agriculture and Forestry University, Fuzhou 350002, China; 3 Department of Agricultural Biotechnology, College of Agriculture and Life Science, Seoul National University, San 56-1, Sillim, Gwanak, Seoul 151-921, Korea

**Keywords:** organic salt, diphenylether, *Aspergillus ochraceopetaliformis*, marine-derived fungi

## Abstract

Ochraceopetalin (**1**), a mixed-biogenetic salt compound and its component **2** were isolated from the culture broths of a marine-derived fungus, *Aspergillus ochraceopetaliformis*. Based on combined spectroscopic and chemical analyses, the structure of **1** was determined to be a sulfonated diphenylether-aminol-amino acid ester guanidinium salt of an unprecedented structural class, while **2** was determined to be the corresponding sulfonated diphenylether. Ochraceopetaguanidine (**3**), the other guanidine-bearing aminol amino acid ester component, was also prepared and structurally elucidated. Compound **1** exhibited significant cytotoxicity against K562 and A549 cells.

## 1. Introduction

Metabolites of polyketide pathways are widely recognized as an important class of fungal natural products due to their significant structural and functional diversities, as well as their wide taxonomical distribution [1,2]. Attributed from the potent bioactivity, several fungal polyketides have been used as lead compounds in the pharmaceutical industry, which culminates to occupy 20% of the major commercial drugs by these compounds, such as lovastatin and griseofulvin [3,4,5]. Tetraketide-derived diphenyl ethers are structurally distinctive from other subclasses of fungal polyketides in terms of their formation by the condensation of two polyketide derivatives [6]. Amidst their wide natural distribution, diphenyl ethers are frequently obtained from marine microorganisms, especially from filamentous fungi of the genera *Aspergillus* and *Penicillium* [7,8]. These compounds possess diverse bioactivities, including antibacterial, cytotoxic, enzyme inhibitory and antiviral activities [9]. Accordingly, these compounds are increasingly applied in cosmetics, pharmaceutical intermediates and chemical products, which in turn have led to extensive studies on their biosynthesis [9]. Recently, a detailed biosynthetic pathway with the gene cluster and key enzyme system was unveiled for diorcinol, which is a representative of fungal diphenyl ethers, in a marine-derived *A. nidulans* strain [6,9].

In the process of searching for bioactive compounds from marine-derived fungi, we recently reported the structures of two new sortase A-inhibitory depsihexapeptides from an *Aspergillus ochraceopetaliformis* fungus, collected from underwater sediment off the coast of Jeju-do, Korea [10]. However, LC–UV and LC–ESI–MS analyses, aided by an in-house spectral library of fungal extracts, revealed the presence of a natural product with distinct structural motifs in a liquid culture broth of this strain. More interestingly, the same analyses of this strain from a rice-based semi-solid culture broth revealed the presence of a structurally related but fragmented compound, prompting an extensive chemical investigation.

**Figure 1:**
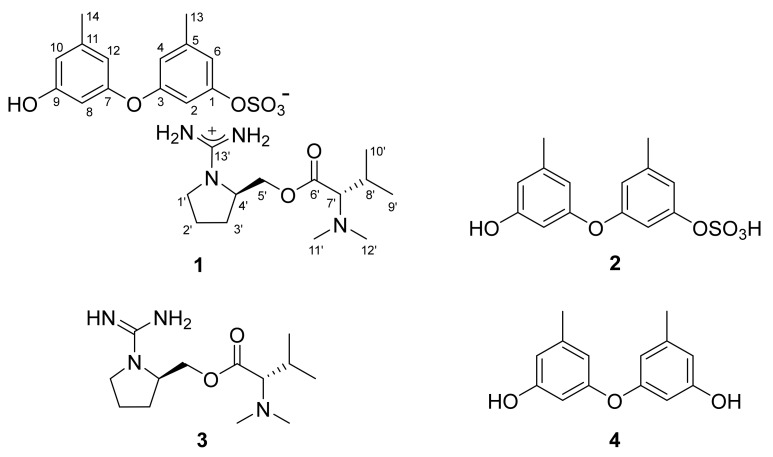
The structures of **1**–**4**.

Herein, we report the structures of ochraceopetalin (**1**), a novel sulfonated diphenylether-aminol-amino acid guanidinium salt and its aromatic component (**2**) (Figure 1). In the process of isolation and structure determination, ochraceopetaguanidine (**3**), a guanidine-bearing aminol amino acid, was structurally elucidated as the other component of **1**. Organic salts coupled with organic counterions are occasionally found in marine organisms: psammaplins, bromotyrosines and dihydroxystyrenes, from an association of *Poecillastra* sp. and *Jaspis* sp. sponges [11,12,13]; suvanines, from a *Coscinoderma mathewsi* sponge [14]; sesterterpene sulfates, from a *Ircinia* sp. sponge [15]; iodotyramine derivative, from the *Didemnum rubem* ascidian [16,17]. To the best of our knowledge, **1** would be the first of this kind to be obtained from marine-derived fungi. Furthermore, its mixed-biogenetic salt formation, that is derived from polyketide and amino acid pathways, would be remarkably unprecedented. Compound **1** exhibited noticeably stronger cytotoxicity toward the human cancer cell lines K562 and A549 than the other compounds.

## 2. Results and Discussion

The molecular formula of compound **1** obtained from the YMM liquid culture broth was deduced to be C_27_H_40_N_4_O_8_S, with 11 degrees of unsaturation, by positive HR–ESI–MS analysis ([M + H]^+^ *m/z* 581.2628, calcd 581.2640). This positive mode MS analysis also gave a highly conspicuous cluster of C_13_H_27_N_4_O_2_ (*m/z* 271.2123, calcd 271.2129), while a similar analysis in negative mode gave a conspicuous cluster of C_14_H_13_O_6_S (*m/z* 309.0433, calcd 309.0427), whose sum corresponded well with the molecular formula of **1**. This phenomenon of significant discrepancy attributed from the opposite MS ionization modes strongly suggested the presence of ionized partial structures, thus the salt nature of **1**. In addition, a strong absorption band was observed in the IR spectrum at 1456 cm^−1^, which corresponds with a sulfate group, as suggested by the MS-indicated sulfur atom.

The ^13^C NMR data of compound **1** showed signals of a carbonyl carbon (δ_C_ 170.5) and thirteen sp^2^ carbons (δ_C_ 160.2–102.7) (Table 1). Based on the combined ^1^H NMR and HSQC data, the chemical shifts and splitting patterns (δ_H_ 6.74–6.16, all s) of corresponding protons were indicative of aromatic moieties. In addition, the odd numbers of sp^2^ carbons suggested the presence of an electron-deficient functionality, such as an imine or guanidine. The NMR data of **1** also contained three sp^3^ methines (δ_C_/δ_H_ 73.3/2.74, 53.0/3.63 and 26.8/1.94), of which the former two carbons would bear nitrogen, according to their carbon and proton chemical shifts. Among the remaining four aliphatic methylene and six methyl signals in the NMR data, the carbon and proton chemical shifts indicated the presence of an oxymethylene (δ_C_/δ_H_ 64.4/4.19 and 4.12), a nitrogenous methylene (δ_C_/δ_H_ 42.7/3.30 and 3.05) and two nitrogenous methyls (δ_C_/δ_H_ 41.0/2.21 × 2).

**Figure 2:**
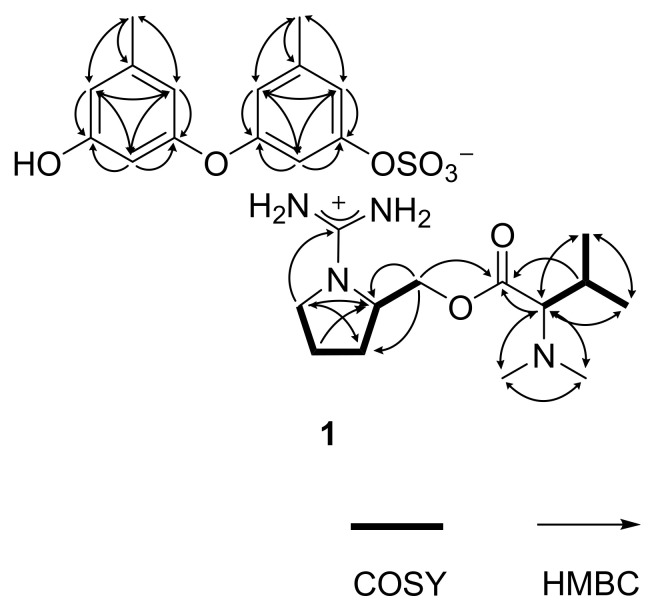
Key correlations of COSY (bold) and HMBC (arrows) experiments for **1**.

The gross structure of **1** was determined by combined ^1^H-^1^H COSY and HMBC experiments. First, all of the aromatic protons (δ_H_ 6.74–6.16, all s) lacked direct proton–proton couplings, placing them at meta- or para-positions to each other. The HMBC correlations of these and two benzylic methyl protons (δ_H_ 2.23, H_3_-13 and δ_H_ 2.18, H_3_-14) with neighboring carbons readily constructed two 1,3-dioxygenated-5-methyl-benzene moieties (C-1–C-6 and C-13, C-7–C-12 and C-14), accounting for eight degrees of unsaturation (Figure 2). Despite the identical proton–carbon correlations, the noticeable differences in both the ^1^H and ^13^C chemical shifts revealed different oxygenated functionalities between aromatic rings. Aided by the negative mode ESI–MS cluster of C_14_H_13_O_6_S and the IR absorption band of the sulfate group at 1456 cm^−1^, the partial structure was interpreted as 9-hydroxy-1-(sulfooxy)-3,7-diphenylether, which is discussed later.

Meanwhile, the COSY data showed the proton spin couplings of an isobutyl group (δ_C_/δ_H_ 73.3/2.74, 26.8/1.94, 19.0/0.82 and 19.5/0.92; C-7′–C-10′) which was confirmed by the HMBC correlations among these. Then, additional proton–carbon correlations of the C-7′ methine and C-8′ methine groups placed a carbonyl (δ_C_ 170.5, C-6′) and two methyl groups (δ_C_/δ_H_ 41.0/2.21 × 2, C-11′ and C-12′) at the adjacent positions. Because the latter methyl groups were determined to be N-CH_3_ by their chemical shifts, nitrogen must be connected to the methyl groups and C-7′. Thus, an *N,N*-dimethyl-valine residue was identified. The COSY data also showed a linear array of four methylenes and a methine (δ_C_/δ_H_ 42.7/3.30 and 3.05, 24.8/1.78 and 1.57, 29.0/1.78 and 1.57, 53.0/3.63 and 64.4/4.19 and 4.12; C-1′–C-5′). Aided by their ^1^H and ^13^C NMR chemical shifts and the crucial HMBC correlation at H_2_-1′/C-4′, a pyrrolidine moiety was identified for C-1′–C-4′. Similarly, the C-5′ methylene was connected to the C-6′ carbonyl by an ester linkage, according to the HMBC correlation at H_2_-5′/C-6′. Thus, a prolinol moiety was defined for the C-1′–C-5′ portion.

The ^13^C NMR data of **1** had a remaining nonprotonated carbon (δ_C_ 160.2), which was placed at the prolinol nitrogen (C-13′) by the crucial HMBC correlation with H_2_-1′. The significant deshielding of this carbon must be attributed to the remaining two nitrogen atoms from the MS data. Thus, in conjunction with the C_13_H_27_N_4_O_2_ cluster observed in the positive ESI–MS analysis, the functionality of C-13′ was assigned to the guanidinium group. Thus, the C-1′–C-13′ portion was defined as 1-(aminoiminomethyl)-prolinol, *N,N*-dimethylvaline ester and the overall structure of **1**, designated as ochraceopetalin, was determined to be a mixed-biogenetic salt.

**Figure 3:**
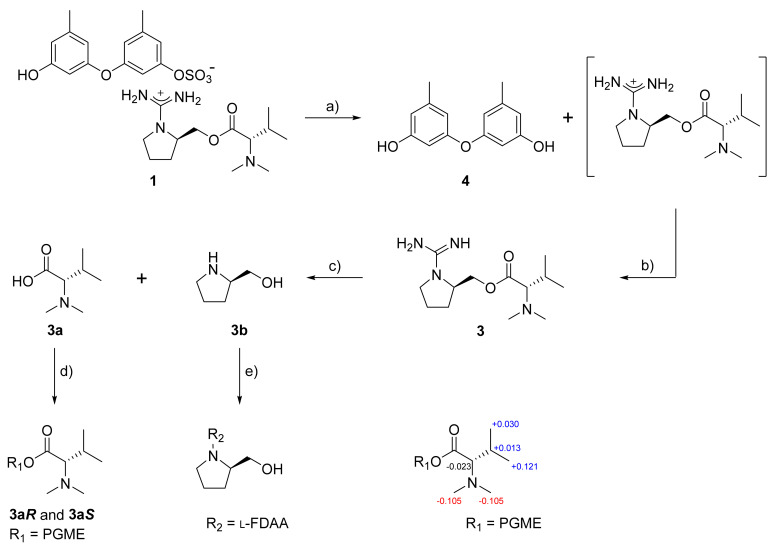
Sequential degradation and configurational assignments of **1**. Reagents and conditions: (**a**) H_2_O, TFA, rt, 3 h; (**b**) NH_4_OH, rt, 10 min; (**c**) NaOH, 100 °C, 3 h; (**d**) PGME, PyBOP, HOBT, *N*-methylmorpholine, rt, 3 h; (**e**) NaHCO_3_, l-FDAA, acetone, 80 °C, 15 min; Δδ values (Δδ = δ*_S_* − δ*_R_*) obtained for (*S*)- and (*R*)-PGME amide derivatives **3a*S*** and **3a*R***.

The proposed structure of **1**, including the salt nature, was confirmed by a series of pH-dependent degradations (Figure 3). When **1** was placed in a mildly acidic solution, a compound separated into an organic layer. A combination of HR–ESI–MS and 1D- and 2D-NMR analyses readily identified this compound as diorcinol (**4**), a well-known 3,3′-oxybis [5-methylphenol] of diverse fungal origins [4,6,8]. Changing the pH of the remaining aqueous solution to basic conditions brought another compound (**3**) into an organic layer. The molecular formula of this compound was deduced to be C_13_H_26_N_4_O_2_ by HR–FAB–MS analysis ([M + H]^+^ *m/z* 271.2135, calcd 271.2129) and the positive mode MS analysis of **3** was identical to that of **1**. Furthermore, the combined 1D- and 2D-NMR data of **3** were identical to those of the cation portion of **1**, which was designated as ochraceopetaguanidine and identified as 1-(aminoiminomethyl)-prolinol, *N,N*-dimethylvaline ester. Thus, the structure of **1** was unambiguously defined as a mixed-biogenetic salt derived from polyketide and amino acid pathways. The sulfate [18,19] and guanidinium salts of **1** was also supported by the comparison of ^13^C NMR data between the simple salts bearing these functionalities and corresponding neutral groups (Figure S27). Compound **1** as a single salt compound was also supported by extensive HPLC analyses, in which **1** was always eluted as single peak under diverse chromatographic conditions (Figure S28).

The production of **1** was further investigated under different culture conditions. After culturing the same strain in a static semi-solid YMM-rice medium, chromatographic separation of the culture broth yielded compound **2**. The molecular formula of **2** was defined to be C_14_H_14_O_6_S by HR–ESI–MS analysis ([M − H]^−^ *m/z* 309.0433, calcd 309.0427), which was identical to the data of **1** in the negative mode MS analysis. Furthermore, the 1D- and 2D-NMR data of **2** were identical to the negative ion portion of **1** (Table 1). In addition, **2** was slowly converted to diorcinol (**4**) during prolonged storage, emphasizing their structural relationship. Thus, the structure of **2** was determined to be 1-(sulfooxy)-diorcinol, a sulfate-bearing diphenyl ether of polyketide origin.

Ochraceopetalin (**1**) and ochraceopetaguanidine (**3**) commonly bear two stereogenic centers in the amino acid portion. For the configurational assignments of these, firstly, a basic hydrolysis of **3** gave *N,N*-dimethylvaline and prolinol. The absolute configuration at C-7′ of the valine residue was assigned using the PGME method [20]. Treatments with (*S*)- and (*R*)-PGME produced the corresponding PGME amides, respectively. The Δδ_H_ values between these unambiguously assigned a d-configuration (Figure 3). For the C-4′ center of prolinol, the absolute configuration was assigned by using the Marfey reaction [21]. After the l-FDAA-prolinol adduct was prepared by condensation with l-FDAA, its HPLC retention time was compared with those of authentic l-FDAA-l-prolinol and l-FDAA-d-prolinol, which resulted in assigning the d-configuration. Thus, both amino acid-derived units of **1** were found to possess d-configurations. In addition, the guanidinium salt of **1** was supported by the ^13^C NMR data of **3c**, the TFA salt of **3**, whose guanidinium bearing C-13′ showed very similar chemical shift with **3** (δ_C_ 161.47 and 160.39 for **3c** and **3**, respectively) (Figure S29). At the same time, the carbon chemical shifts of C-13′ of **1** and **3** also showed similarity with 0.15 ppm and 0.13 ppm differences in DMSO-*d*_6_ (δ_C_ 160.24 and δ_C_ 160.39 for **1** and **3**, respectively) and MeOH-*d*_4_ solvent (δ_C_ 161.95 and δ_C_ 162.08 for **1** and **3**, respectively), respectively (Figures S30–S31, Table S2).

In addition to the structure determination, the structure of **1** and **2** led to an interesting argument. Diorcinol (**4**) is widely recognized as a natural product of diverse fungi, such as *Aspergillus versicolor* GH-2 [22], *A. tennesseensis* [23] and *Cordyceps* sp. [24]. The detailed biosynthesis via orsellinic acid, including the gene cluster and enzymatic reactions, was recently determined for a marine-derived *A. nidulans* [6,9]. However, our results of finding **1** and **2** consecutively (eight times of cultivation) raise the possibility that, in the case of this strain (strain number FJ120) of *A. ochraceopetaliformis*, the natural product of the fungus is not diorcinol (**4**), but 1-(sulfooxy)-diorcinol (**2**), or its salt (**1**). Alternatively, it would be possible that naturally produced **4** was sulfonated to **1** or **2** by a biotransformation during the cultivation. It would be also possible to transform **1** by an abiotic process during the cultivation or isolation procedures. To clarify this, time-dependent cultivation and ESI–MS analyses of the broths revealed two interesting phenomena. The first is that **1** and **2** were independently produced after 8 and 6 weeks of cultivation, respectively, under given culturing conditions, while the production of **4** was undetected by LC–ESI–MS analysis (Figures S24 and S25). The second interesting phenomenon is that distinct products according to the culturing conditions could be attributed to the differences in pH in the culture media, **1**, pH 9.0, and **2**, pH 5.0. This question of biosynthesis of diorcinol derivatives would be fully answered only by the extensive biosynthetic study on this strain.

Compounds **1**–**4** were tested using a number of bioassays. In cytotoxicity tests, all four compounds were either significantly inhibitory (**1**, IC_50_ 9.5 and 6.8 μM), or less active (**2**–**4**, IC_50_ 11–25 μM) against the human cancer cell lines K562 and A549 (Figure S26 and Table S1). Although its cytotoxicity was not remarkable, **1** was noticeably more potent than its components **2**–**4**. These microbial compounds failed to inhibit both Gram-positive and Gram-negative bacteria and pathogenic fungi (MIC > 128 μg/mL). They were also inactive against the microbial enzymes sortase A (SrtA), a transpeptidase responsible for the anchoring of surface proteins to the cell wall envelope of Gram-positive bacteria and isocitrate lyase (ICL), a key enzyme in the glyoxylate cycle.

In summary, ochraceopetalin (**1**), a mixed-biogenetic salt compound and its component **2** were isolated from a marine-derived fungus *Aspergillus ochraceopetaliformis*. Based on the results of combined spectroscopic and chemical analyses, the structure of **1** was determined to be a sulfonated diphenylether-aminol-amino acid guanidinium of an unprecedented structural class. Compounds **2** and **3**, obtained from the different culturing condition and chromatographic processes, respectively, were defined to be the corresponding components of **1**, 1-(sulfooxy)-diorcinol and 1-(aminoiminomethyl)-prolinol, *N,N*-dimethylvaline ester, respectively. Compound **1** exhibited significant cytotoxicity against K562 and A549 cells.

## 3. Materials and Methods

### 3.1. General Experimental Procedures

Optical rotations were measured on a JASCO P1020 polarimeter (Jasco, Tokyo, Japan) using a 1 cm cell. UV spectra were acquired with a Hitachi U-3010 spectrophotometer (Hitachi High-Technologies, Tokyo, Japan). IR spectra were recorded on a JASCO 4200 FT-IR spectrometer (Jasco, Tokyo, Japan) using a ZnSe cell. ^1^H and ^13^C NMR spectra were measured in DMSO-*d*_6_, CDCl_3_, or MeOH-*d*_4_ solutions on Bruker Avance -400, -500, -600, or -800 instruments (Bruker, Billerica, MA, USA), with solvent peaks at δ_H_ 2.50/δ_C_ 39.50, δ_H_ 7.26/δ_C_ 77.16 and δ_H_ 3.31/δ_C_ 49.00, respectively, as their internal standards. High-resolution ESI mass spectrometric data were obtained at the National Instrumentation Center for Environmental Management (Seoul, Korea) and were acquired using an AB Sciex 5600 QTOF HR-MS instrument (Sciex, Washington, DC, USA). High-resolution FAB mass spectrometric data were obtained at the Korea Basic Science Institute (Daegu, Korea) and were acquired using a JEOL JMS 700 mass spectrometer (Jeol, Tokyo, Japan) with *meta*-nitrobenzyl alcohol (NBA) as the matrix. Low-resolution ESI–MS data were recorded on an Agilent Technologies 6130 quadrupole mass spectrometer with an Agilent Technologies 1200 series HPLC. Semi-preparative and analytical HPLC separations were performed on a Spectrasystem p2000 equipped with a Spectrasystem RI-150 refractive index detector and a UV-Vis-151 detector (Gilson, Middleton, WI, USA). All the solvents used were of spectroscopic grade or distilled from glass prior to use.

### 3.2. Fungal Material

The fungal strain *Aspergillus ochraceopetaliformis* (strain number FJ120) was isolated from marine sediments collected from Jeju-do, Korea, in July 2007 [10]. The isolate was identified using standard molecular biology protocols by DNA amplification and sequencing of the ITS region. Genomic DNA extraction was performed using Intron’s i-genomic BYF DNA Extraction Mini Kit, according to the manufacturer’s protocol. The nucleotide sequence was deposited in the GenBank database under accession number KF384187. The 18S rDNA sequence of this strain exhibited 100% identity (588/588) with that of *Aspergillus ochraceopetaliformis* strain RKI08-134 (GenBank accession number FJ797698).

### 3.3. Fermentation

The fungal strain was cultured on solid YPG media (5 g of yeast extract, 5 g of peptone, 10 g of glucose, 24.8 g of sea salt and 16 g of agar in 1 L of distilled water) for 7 days. An agar plug (1 cm × 1 cm) was inoculated in 250 mL flask containing 100 mL of YPG media. After 7 days of growth, 10 mL of each culture was transferred to 2.8 L Fernbach flasks containing YMM media (5 g of yeast extract, 5 g of malt extract, 10 g of mannitol and 24.8 g of sea salt in 1000 mL of distilled water). In total, 30 L of YMM media was prepared and cultivated under static conditions for 8 weeks at 30 °C.

The large-scale cultivation of YPG-based seed culture was also performed on semi-solid YMM-rice media (1 g of yeast extract, 1 g of malt extract, 2 g of mannitol and 200 g of rice in 200 mL of artificial seawater) in 2.8 L Fernbach flasks at 30 °C in the static condition for 6 weeks.

### 3.4. Extraction and Isolation

The entire culture was filtered and extracted with EtOAc (20 L × 3). The solvent was evaporated in vacuo to afford a brown organic gum (4.3 g). The extract was separated by C_18_ reversed-phase vacuum flash chromatography using sequential mixtures of H_2_O and MeOH (five fractions of H_2_O-MeOH, gradient from 80:20 to 0:100), acetone and, finally, EtOAc as the eluents. Based on the results of ^1^H NMR and LR–ESI–MS analyses, the fraction eluted with H_2_O-MeOH 40:60 (850 mg) was subjected to semi-preparative reversed-phase HPLC (YMC-ODS-A column, 250 × 10 mm; H_2_O-MeOH, 50:50, 1.7 mL/min), affording compound **1** (*t*_R_ = 29.1 min). Compound **1** was further purified by analytical HPLC (YMC-ODS-A column, 250 × 4.6 mm; H_2_O-MeCN, 75:25, 0.7 mL/min; *t*_R_ = 22.0 min; 11.1 mg).

The broth (4.2 kg) from semi-solid YMM-rice media was extracted with methanol (21 L × 3) and dichloromethane (21 L × 3). The combined extracts (111.1 g) were dried in vacuo and partitioned between H_2_O (84.6 g) and *n*-BuOH (26.4 g). The organic layer was repartitioned between H_2_O-MeOH (15:85, 14.2 g) and *n*-hexane (12.2 g). The H_2_O-MeOH layer was evaporated to obtain an organic extract (14.2 g). The extract was fractionated by C_18_ reversed-phase vacuum flash chromatography using a sequential mixture of MeCN and H2O as eluents (H_2_O-MeCN, from 80:20 to 50:50), MeOH, acetone and, finally, EtOAc. On the basis of the result of ^1^H NMR and LC-MS profile, the fraction (1240 mg) eluted with H_2_O-MeCN (70:30) was purified by semi-preparative reversed-phase HPLC (YMC-ODS-A column, 250 × 10 mm; H_2_O-MeOH, 55:45, 1.7 mL/min) to yield compound **2** (*t*_R_ = 34.5 min). Compound **2** was further purified by analytical HPLC (YMC-ODS-A column, 250 × 4.6 mm; H_2_O-MeCN, 80:20, 0.7 mL/min; *t*_R_ = 15.6 min; 13.7 mg).

Ochraceopetalin (**1**): white amorphous solid; [α]***25D*** -5 (*c* 0.4, MeOH); UV (MeOH) λ_max_ (log ε) 209 (3.17), 275 (2.01) nm; IR (ZnSe) ν_max_ 3347 (br), 2946, 1456, 1031 cm^−1^; ^1^H and ^13^C NMR data, Table 1; HR–ESI–MS *m/z* 581.2628 [M + H]^+^ (calcd for C_27_H_41_N_4_O_8_S, 581.2640).

1-(Sulfooxy)-diorcinol (**2**): yellow amorphous solid; UV (MeOH) λ_max_ (log ε) 210 (3.16), 275 (2.19) nm; IR (ZnSe) ν_max_ 3359 (br), 2833, 1455, 1033 cm^−1^; ^1^H and ^13^C NMR data, Table 1; HR–ESI–MS *m/z* 309.0437 [M − H]^−^ (calcd for C_14_H_13_O_6_S, 309.0438).

### 3.5. pH-Dependent Hydrolysis of Compound **1**

Firstly, compound **1** (9.7 mg) was dissolved in water (0.05% TFA, 5 mL). The solution was stood at room temperature for 3 h. The aqueous solution was partitioned with *n*-BuOH (5 mL) through a separate funnel. Removal of *n*-BuOH in vacuo yielded pure compound **4** (3.5 mg). To the aqueous fraction, 0.05% of NH_4_OH (5 mL) was added. After removing water, purification by analytical HPLC (YMC-ODS-A column, 250 × 4.6 mm; 0.7 mL/min; H_2_O-MeCN, 70:30) afforded compound **3** (*t*_R_ = 21.1 min; 4.2 mg).

Ochraceopetaguanidine (**3**): white amorphous solid; [α]***25D*** -4 (*c* 0.4, MeOH); UV (MeOH) λ_max_ (log ε) 205 (2.39) nm; IR (ZnSe) ν_max_ 3362 (br), 2360, 1636 cm^−1^; ^1^H and ^13^C NMR data, Table 1; HR-FAB-MS *m/z* 271.2135 [M + H]^+^ (calcd for C_13_H_27_N_4_O_2_, 271.2129).

Diorcinol (**4**): colorless gum; ^1^H and ^13^C NMR spectra, Figures S17 and S18; HR–ESI–MS *m/z* 231.1012 [M + H]^+^ (calcd for C14H15O3, 231.1016).

### 3.6. Basic Hydrolysis of Compound ***3***

Compound **3** (3.5 mg) was dissolved in 1 N NaOH (1 mL) and the solution was stirred at 100 °C for 3 h. The separation by analytical HPLC (YMC-ODS-A column, 250 × 4.6 mm; 0.7 mL/min; H_2_O-MeCN, 65:35) afforded compounds **3a** (*N*,*N*-dimethylvaline, *t*_R_ = 15.4 min; 2.2 mg) and **3b** (prolinol, *t*_R_ = 23.8 min; 2.8 mg) as pure compounds.

*N,N*-Dimethylvaline (**3a**): white amorphous solid; ^1^H NMR (DMSO-*d*_6_, 400 MHz) δ_H_ 3.16 (1H, br s, 6′-OH), 2.66 (1H, d, *J* = 9.1 Hz, H-7′), 2.29 (6H, s, H-11′, H-12′), 1.90 (1H, dhep, *J* = 9.1, 6.8 Hz, H-8′), 0.92 (3H, d, *J* = 6.6 Hz, H-10′), 0.83 (3H, d, *J* = 6.6 Hz, H-9′); LR–ESI–MS *m/z* 146.2 [M + H]^+^.

Prolinol (**3b**): pale yellow oil; ^1^H NMR (CDCl_3_, 400 MHz) δ_H_ 3.81 (2H, OH, NH), 3.37 (1H, dd, *J* = 11.0, 4.0 Hz, H-5′), 3.20 (1H, dd, *J* = 11.0, 7.0 Hz, H-5′), 3.02 (1H, m, H-4′), 2.71 (1H, m, H-1′), 2.68 (1H, m, H-1′), 1.63–1.52 (3H, m, H-2′,3′), 1.22 (1H, m, H-2′); LR–ESI–MS *m/z* 102.1 [M + H]^+^.

### 3.7. Preparation of (S)- and (R)-PGME Amides of ***3a***

To a dry DMF solution (500 μL) of compound **3a** (0.5 mg, 6.9 mM) and (*S*)-PGME (1.5 mg, 33 mM), PyBOP (8.5 mg, 33 mM), HOBT (2.2 mg, 33 mM) and *N*-methylmorpholine (100 μL) were added. After stirring the mixture for 3 h at room temp, a 5% HCl solution and EtOAc were added to the reaction mixture. The EtOAc layer was subsequently washed with saturated NaHCO_3_ solution and brine. The organic layer was dried over anhydrous Na_2_SO_4_. After removing the solvent under vacuum, the residue was purified by reversed-phase HPLC (YMC-ODS column, 250 × 4.6 mm; H_2_O-MeCN, 25:75) to give (*S*)-PGME amide **3a*S*** (0.2 mg). Compound **3a*R*** (0.2 mg), the (*R*)-PGME amide of **3a**, was prepared from (*R*)-PGME in a similar fashion. The molecular formulae of **3a*S*** and **3a*R*** were confirmed as C_16_H_24_N_2_O_3_ based on LR–ESI–MS data.

(*S*)-PGME amide of **3a** (**3a*S***): white amorphous solid; ^1^H NMR (CD_3_OD, 800 MHz) δ_H_ 7.393–7.338 (5H, m, PGME-Ar), 5.495 (1H, s, PGME-H-1), 3.697 (3H, s, PGME-OMe), 2.716 (1H, d, *J* = 9.0 Hz, H-7′), 2.240 (6H, s, H-11′, H-12′), 2.050 (1H, dhep, *J* = 9.3, 6.7 Hz, H-8′), 0.975 (3H, d, *J* = 6.7 Hz, H-10′), 0.931 (3H, d, *J* = 6.7 Hz, H-9′); LR–ESI–MS *m/z* 293.4 [M + H]^+^.

(*R*)-PGME amide of **3a** (**3a*R***): white amorphous solid; ^1^H NMR (CD_3_OD, 800 MHz) δ_H_ 7.373–7.335 (5H, m, PGME-Ar), 5.481 (1H, s, PGME-H-1), 3.704 (3H, s, PGME-OMe), 2.739 (1H, d, *J* = 9.2 Hz, H-7′), 2.345 (6H, s, H-11′, H-12′), 2.037 (1H, dhep, *J* = 9.4, 6.7 Hz, H-8′), 0.945 (3H, d, *J* = 6.7 Hz, H-10′), 0.810 (3H, d, *J* = 6.7 Hz, H-9′); LR–ESI–MS *m/z* 293.4 [M + H]^+^.

### 3.8. Marfey’s Analysis of ***3b***

Compound **3b** (0.5 mg) was dissolved in 12 N HCl (0.5 mL) and heated at 110 °C for 16 h. The solution and traces of HCl were removed by repeated drying under vacuum with distilled water. To the hydrolysate, 1 N NaHCO_3_ (100 µL) and 1% l- or d-FDAA (50 µL) in acetone were added. The mixture was stirred at 80 °C for 15 min. After quenching the reaction by the addition of 2 N HCl (50 µL), the residue was analyzed using HPLC with an analytical column (YMC-ODS column, 250 × 4.6 mm; H_2_O-MeCN, 60:40). As standard compounds, l- and d-prolinol were also prepared using this method. The retention times of the l-FDAA-derivatized l-prolinol was 19.686 min and that of l-FDAA-derivatized d-prolinol was 20.160 min. The retention time of the l-FDAA-derivatized **3b** was 20.222 min, leading to assignment of the d-configuration.

### 3.9. Antibacterial, Antifungal, Enzyme-Inhibitory and Cytotoxic Assays

#### 3.9.1. Antibacterial Activity Assay

To investigate the antibacterial activity of isolated compounds, a series of minimal inhibitory concentration (MIC) tests was performed according to the Clinical and Laboratory Standards Institute (CLSI) guide methods [25]. Three species of Gram-positive bacteria (*S. aureus* strain Newman, *E. faecalis* ATCC19433 and *E. faecium* ATCC19434) and three species of Gram-negative bacteria (*K. pneumoniae* ATCC10031, *S. enterica* ATCC14028 and *E. coli* ATCC25922) were selected as test strains. The MIC of each test compound against six bacterial strains was determined in liquid culture using Mueller–Hinton broth (inoculum concentration, 5 × 10^4^ cfu/mL) with the compound (concentration range of 0.06–128 µg/mL) after 24 h incubation at 37 °C. Ampicillin and tetracycline were used as reference compounds.

#### 3.9.2. Antifungal Activity Assay

The antifungal activity of isolated compounds was estimated against *C. albicans* ATCC10231, *A. fumigatus* HIC6094, *T. rubrum* NBRC9185 and *T. mentagrophytes* IFM40996 according to the guidelines in CLSI document M38 [26]. The growth of test fungi was monitored in RPMI 1640 broth (inoculum concentration: 10^4^ cells/mL) with the compound concentration range of 0.06–128 µg/mL. Amphotericin B was used as a positive control.

#### 3.9.3. ICL Inhibition Assay

The purification of recombinant ICL from the genomic DNA of *C. albicans* ATCC10231 and the evaluation of the effect of isolated compounds on ICL were carried out by the method described previously [27]. The formation of glyoxylate phenylhydrazone from isocitrate and phenylhydrazine substrates was monitored spectrophotometrically at 324 nm. 3-Nitropropionic acid was used as a positive control.

#### 3.9.4. SrtA Inhibition Assay

The preparation of recombinant SrtA from *S. aureus* ATCC6538p and the evaluation of the effect of isolated compounds on SrtA were performed according to a previously described procedure [27]. The enzyme reaction was carried out at 37 °C for 1 h with 300 µL of buffer (50 mM Tris–HCl, 150 mM NaCl, 5 mM CaCl_2_, pH 7.5), 55 µg of purified SrtA, 0.75 µg of synthetic peptide dabcyl-QALPETGEE-edans and test samples at various concentrations. The increase in the fluorescence intensity was recorded by a fluorescence spectrophotometer (excitation, 350 nm; emission, 495 nm). Curcumin and berberine chloride were used as positive controls.

#### 3.9.5. Cytotoxic Assay

The effects of compounds **1**–**4** on cell viability were analyzed by MTT assay [27]. A549 (lung cancer) and K562 (leukemia) cells were purchased from the Korean Cell Line Bank (KCLB), Seoul, Korea. Both cell lines were cultured in RPMI-1640 medium with L-glutamine, 10% fetal bovine serum and 1% penicillin/streptomycin. All the cells were cultured at 37 °C in a humidified atmosphere with 5% CO_2_. For the assay, each cell (5 × 10^4^ cells/mL) was seeded in 96-well plates (100 µL/well). After 24 h, they were treated with various concentrations of compounds **1**–**4**. After 24 h of compound treatment, MTT (250 µg/mL) was added to each well and incubated for 4 h. The formazan product was dissolved with 250 µL of DMSO. Absorbances of each well were detected at the wavelength of 595 nm using an ELISA microplate reader (BioTek, Winooski, VT, USA) to determine the cell viability by quantifying the production of formazan. The IC_50_ values were calculated using a nonlinear regression analysis (percent survival versus concentration). Doxorubicin was used as a positive control.

## Figures and Tables

**Table 1:** The ^13^C and ^1^H NMR data of compounds **1**–**3** ^a,b^ (δ_H_ and δ_C_ in ppm).

No.	1		2		3	
	δ_C_, Type	δ_H_ (*J* in Hz)	δ_C_, Type	δ_H_ (*J* in Hz)	δ_C_, Type	δ_H_ (*J* in Hz)
1	154.5, C		154.5, C			
2	108.1, CH	6.61, s	108.1, CH	6.61, s		
3	156.6, C		156.7, C			
4	113.9, CH	6.49, s	113.9, CH	6.49, s		
5	139.3, C		139.3, C			
6	115.8, CH	6.74, s	116.0, CH	6.74, s		
7	157.6, C		157.6, C			
8	102.7, CH	6.16, s	102.8, CH	6.16, s		
9	158.4, C		158.6, C			
10	109.8, CH	6.25, s	109.9, CH	6.25, s		
11	140.1, C		140.1, C			
12	111.2, CH	6.34, s	111.2, CH	6.34, s		
13	21.0, CH_3_	2.23, s	21.1, CH_3_	2.23, s		
14	21.1, CH_3_	2.18, s	21.1, CH_3_	2.18, s		
9-OH		9.47, s		9.49, br s		
1′	42.7, CH_2_	3.30, m, 3.05, m			42.7, CH_2_	3.30, m, 3.05, m
2′	24.8, CH_2_	1.78, m, 1.57, m			24.8, CH_2_	1.78, m, 1.57, m
3′	29.0, CH_2_	1.78, m, 1.57, m			29.0, CH_2_	1.78, m, 1.57, m
4′	53.0, CH	3.63, d (7.0)			52.9, CH	3.63, m
5′	64.4, CH_2_	4.19, dd (11.5, 5.0)			64.6, CH_2_	4.19, dd (11.5, 4.5)
		4.12, dd (11.5, 6.5)				4.12, dd (11.5, 6.5)
6′	170.5, C				170.4, C	
7′	73.3, CH	2.74, d (10.5)			73.2, CH	2.74, d (10.5)
8′	26.8, CH	1.94, dhep (10.5, 6.5)			26.8, CH	1.94, dhep (10.5, 6.5)
9′	19.0, CH_3_	0.82, d (6.5)			19.2, CH_3_	0.82, d (6.5)
10′	19.5, CH_3_	0.92, d (6.5)			19.2, CH_3_	0.92, d (6.5)
11′	41.0, CH_3_	2.21, s			41.0, CH_3_	2.21, s
12′	41.0, CH_3_	2.21, s			41.0, CH_3_	2.21, s
13′	160.2, C				160.4, C	

^a^ Data were obtained in DMSO-*d*_6_. ^b^ Data were measured at 125/500 (**1**) and 150/600 (**2** and **3**) MHz for δ_C_/δ_H_, respectively.

## Supplementary Materials

The following are available online at https://www.mdpi.com/article/10.3390/md19080413/s1, Figures S1–S5, S7–S18, S30–S31: annotated 1D NMR and selected 2D NMR spectra of **1**–**4**, Figure S6: HR–ESI–MS of **1**, Figures S19–S22: ^1^H NMR spectra of **3a**, **3b**, **3a*S*** and **3a*R***, Figure S23: The LC/MS chromatogram data of **3b-l-FDAA** adduct, Figures S24–S25: The time-scale LC–MS analysis of YMM liquid media extracts and YMM-rice-semi-solid media extracts, Figure S26: MTT assay against K562 and A549 cancer cells, Table S1: Results of cytotoxicity tests, Figure S27: The ^13^C NMR (25 MHz, D_2_O) spectra of guanidine and guanidine hydrochloride from Scifinder, Figure S28: The HPLC analysis of **1**, Figure S29: ^13^C NMR spectrum of **3c**, Table S2: The ^13^C NMR data of **1**–**3** and **3c**.

## References

[1] Hussain H., Al-Sadi A.M., Schulz B., Steinert M., Khan A., Green I.R., Ahmed I. (2017). A fruitful decade for fungal polyketides from 2007 to 2016: Antimicrobial activity, chemotaxonomy and chemodiversity. Future Med. Chem..

[2] Aldholmi M., Marchand P., Ourliac-Garnier I., Le Pape P., Ganesan A. (2019). A decade of antifungal leads from natural products: 2010–2019. Pharmaceuticals.

[3] Weissman K.J., Leadlay P.F. (2005). Combinatorial biosynthesis of reduced polyketides. Nat. Rev. Microbiol..

[4] Albers-Schönberg G., Joshua H., Lopez M.B., Hensens O.D., Springer J.P., Chen J., Ostrove S., Hoffman C.H., Alberts A.W., Patchett A.A. (1981). Dihydromevinolin, a potent hypocholesterolemic metabolite produced by *Aspergillus terreus*. J. Antibiot..

[5] Oxford A.E., Raistrick H., Simonart P. (1939). Studies in the biochemistry of micro-organisms: Griseofulvin, C(17)H(17)O(6)Cl, a metabolic product of *Penicillium griseo-fulvum* Dierckx. Biochem. J..

[6] Sanchez J.F., Chiang Y.-M., Szewczyk E., Davidson A.D., Ahuja M., Oakley C.E., Bok J.W., Keller N., Oakley B.R., Wang C.C.C. (2010). Molecular genetic analysis of the orsellinic acid/F9775 gene cluster of *Aspergillus nidulans*. Mol. Biosyst..

[7] Xu X., Yang H., Xu H., Yin L., Chen Z., Shen H. (2018). Diphenyl ethers from a marine-derived isolate of *Aspergillus* sp. CUGB-F046. Nat. Prod. Res..

[8] Zhang Y., Li X.-M., Shang Z., Li C.-S., Ji N.-Y., Wang B.-G. (2012). Meroterpenoid and diphenyl ether derivatives from *Penicillium* sp. MA-37, a fungus isolated from marine mangrove rhizospheric soil. J. Nat. Prod..

[9] Feng C., Wei Q., Hu C., Zou Y. (2019). Biosynthesis of diphenyl ethers in fungi. Org. Lett..

[10] Hwang J.-Y., Lee J.-H., Park S.C., Lee J., Oh D.-C., Oh K.-B., Shin J. (2019). New peptides from the marine-derived fungi *Aspergillus allahabadii* and *Aspergillus ochraceopetaliformis*. Mar. Drugs.

[11] Park Y., Liu Y., Hong J., Lee C.-O., Cho H., Kim D.-K., Im K.S., Jung J.H. (2003). New bromotyrosine derivatives from an association of two sponges, *Jaspis wondoensis* and *Poecillastra wondoensis*. J. Nat. Prod..

[12] Shinde P.B., Lee Y.M., Dang H.T., Hong J., Lee C.-O., Jung J.H. (2008). Cytotoxic bromotyrosine derivatives from a two-sponge association of *Jaspis* sp. and *Poecillastra* sp.. Bioorg. Med. Chem. Lett..

[13] Chang Y.H., Shin D., Na Z., Lee H.-S., Kim D.-D., Oh K.-B., Shin J. (2008). Dihydroxystyrene metabolites from an association of sponges *Poecillastra wondoensis* and *Japis* sp.. J. Nat. Prod..

[14] Kimura J., Ishizuka E., Nakao Y., Yoshida W.Y., Scheuer P.J., Kelly-Borges K. (1998). Isolation of 1-methylherbipoline salts of halisulfate-1 and of suvanine as serine protease inhibitors from a marine sponge, *Coscinoderma mathewsi*. J. Nat. Prod..

[15] Wright A.E., McCarthy P.J. (1989). Sulfircin: A new sesterterpene sulfate from a deep-water sponge of the genus *Ircinia*. J. Org. Chem..

[16] Solano G., Motti C.A., Jaspars M. (2009). New iodotyramine derivatives from *Didemnum rubeum*. Tetrahedron.

[17] Carroll A.R., Copp B.R., Davis R.A., Keyzers R.A., Prinsep M.R. (2021). Marine natural products. Nat. Prod. Rep..

[18] Huibers M., Manuzi A., Rutjes F.P.J.T., van Delft F.L.J. (2006). A Sulfitylation−Oxidation Protocol for the Preparation of Sulfates. Org. Chem..

[19] Brandstorm A., Strandlund G., Lagerstrom P.-O. (1980). Kinetic Studies of the Sulfonation of 2-*tert*-Butylphenol with Chlorosulfonic Acid. Acta Chem. Scand..

[20] Yabuuchi T., Kusumi T. (2000). Phenylglycine methyl ester, a useful tool for absolute configuration determination of various chiral carboxylic acids. J. Org. Chem..

[21] Tsuda M., Sasaki M., Mugishima T., Komatsu K., Sone T., Tanaka M., Mikami Y., Kobayashi J. (2005). Scalusamides A-C, new pyrrolidine alkaloids from the marine-derived fungus *Penicillium citrinum*. J. Nat. Prod..

[22] Hua S.-S., Jiang N., Wanga X.-L., Chen C.-J., Fan J.-Y., Wurin G., Ge H.-M., Tan R.-X., Jiao R.-H. (2015). Prenylated diphenyl ethers from the mantis-associated fungus *Aspergillus versicolor* GH-2. Tetrahedron Lett..

[23] Li Z.-X., Wang X.-F., Ren G.-W., Yuan X.-L., Deng N., Ji G.-X., Li W., Zhang P. (2018). Prenylated diphenyl ethers from the marine algal-derived endophytic fungus *Aspergillus tennesseensis*. Molecules.

[24] Bunyapaiboonsri T., Yoiprommarat S., Intereya K., Kocharin K. (2007). New diphenyl ethers from the insect pathogenic fungus *Cordyceps* sp. BCC 1861. Chem. Pharm. Bull..

[25] Oh K.-B., Lee J.H., Chung S.-C., Shin J., Shin H.J., Kim H.-K., Lee H.-S. (2008). Antimicrobial activities of the bromophenols from the red alga *Odonthalia corymbifera* and some synthetic derivatives. Bioorg. Med. Chem. Lett..

[26] Lee H.-S., Lee T.-H., Yang S.H., Shin H.J., Shin J., Oh K.-B. (2007). Sesterterpene sulfates as isocitrate lyase inhibitors from tropical sponge *Hippospongia* sp.. Bioorg. Med. Chem. Lett..

[27] Kwon O.-S., Kim C.-K., Byun W.S., Oh J., Lee Y.-J., Lee H.-S., Sim C.J., Oh D.-C., Lee S.K., Oh K.-B. (2018). Cyclopeptides from the sponge *Stylissa flabelliformis*. J. Nat. Prod..

