# Supplementary material for: Ochraceopetalin, a Mixed-Biogenetic Salt of Polyketide and Amino Acid Origins from a Marine-Derived Aspergillus ochraceopetaliformis Fungus
Source: Mar Drugs. 2021 Jul 26;19(8):413. doi: 10.3390/md19080413 (PMC8401040; doi:10.3390/md19080413)
Supplement: Supplementary file 1 [file marinedrugs-19-00413-s001.zip › marinedrugs-1307422-supplementary.pdf]

Supporting Information

Ochraceopetalin, a Mixed-Biogenetic Salt of Polyketide and Amino Acid Origins from a Marine-Derived *Aspergillus ochraceopetaliformis* Fungus

Sung Chul Park [1,†], Jung-Ho Lee [1,†], Ji-Yeon Hwang [1], Oh-Seok Kwon [1], Lijuan Liao [1,2], Dong-Chan Oh [1], Ki-Bong Oh [3,*] and Jongheon Shin [1,*]

1 Natural Products Research Institute, College of Pharmacy, Seoul National University, San 56-1, Sillim, Gwanak, Seoul 151-742, Korea; sungchulpark@snu.ac.kr (S.C.P.); anilove@snu.ac.kr (J.-H..L.); yahyah7@snu.ac.kr (J.-Y.H.); ideally225@snu.ac.kr (O.-S.K.); lxycxf@snu.ac.kr (L.L.); dongchanoh@snu.ac.kr (D.-C.O.); shinj@snu.ac.kr (J.S.)

2 College of Life Sciences, Fujian Agriculture and Forestry University, Fuzhou 350002, China; lxycxf@snu.ac.kr (L.L.)

3 Department of Agricultural Biotechnology, College of Agriculture and Life Science, Seoul National University, San 56-1, Sillim, Gwanak, Seoul 151-921, Korea; ohkibong@snu.ac.kr (K.-B.O.)

* Correspondence: shinj@snu.ac.kr (J.S.); ohkibong@snu.ac.kr (K.-B.O.); Tel.: +82-2-880-2484 (J.S.); +82-2-880-4646 (K.-B.O.)

† Equal contribution.

Contents

Figure S1. The ^{1}H NMR (500 MHz, DMSO-d_6) spectrum of **1** S3

Figure S2. The ^{13}C NMR (125 MHz, DMSO-d_6) spectrum of **1** S4

Figure S3. The COSY (500 MHz, DMSO-d_6) spectrum of **1** S5

Figure S4. The HSQC (500 MHz, DMSO-d_6) spectrum of **1** S6

Figure S5. The HMBC (500 MHz, DMSO-d_6) spectrum of **1** S7

Figure S6. HR-ESI-MS data of **1** S8

Figure S7. The ^{1}H NMR (600 MHz, DMSO- d_6) spectrum of **2** S9

Figure S8. The ^{13}C NMR (150 MHz, DMSO- d_6) spectrum of **2** S10

Figure S9. The COSY (600 MHz, DMSO- d_6) spectrum of **2** S11

Figure S10. The HSQC (600 MHz, DMSO- d_6) spectrum of **2** S12

Figure S11. The HMBC (600 MHz, DMSO- d_6) spectrum of **2** S13

Figure S12. The ^{1}H NMR (600 MHz, DMSO-d_6) spectrum of **3** S14

Figure S13. The ^{13}C NMR (150 MHz, DMSO-d_6) spectrum of **3** S15

Figure S14. The COSY (600 MHz, DMSO-d_6) spectrum of **3** S16

Figure S15. The HSQC (600 MHz, DMSO-d_6) spectrum of **3** S17

Figure S16. The HMBC (600 MHz, DMSO-d_6) spectrum of **3** S18

Figure S17. The ^{1}H NMR (400 MHz, $CDCl_3$) spectrum of **4** S19

Figure S18. The ^{13}C NMR (100 MHz, $CDCl_3$) spectrum of **4** S20

Figure S19. The ^{1}H NMR (400 MHz, DMSO-d_6) spectrum of **3a** S21

Figure S20. The ^{1}H NMR (400 MHz, $CDCl_3$) spectrum of **3b** S22

Figure S21. The ^{1}H NMR (800 MHz, Methanol-d_4) spectrum of **3a*S*** S23

Figure S22. The ^{1}H NMR (800 MHz, Methanol-d_4) spectrum of **3a*R*** S24

Figure S23. The LC/MS chromatogram data of **3b-L-FDAA** adduct S25

Figure S24. The time-scale LC-MS analysis of YMM liquid media extracts S25

Figure S25. The time-scale LC-MS analysis of YMM-rice semi-solid media extracts S26

Figure S26. The MTT assay against K562 and A549 cancer cells S27

Table S1. Results of cytotoxicity tests S27

Figure S27. The ^{13}C NMR (25 MHz, D_2O) spectra of guanidine and guanidine hydrochloride S28

Figure S28. The HPLC analysis of **1** S29

Figure S29. The ^{13}C NMR (100 MHz, DMSO-d_6) spectrum of **3c** S30

Figure S30. The ^{13}C NMR (125 MHz, MeOH-d_4) spectrum of **1** S31

Figure S31. The ^{13}C NMR (200 MHz, MeOH-d_4) spectrum of **3** S32

Table S2. The ^{13}C NMR data of **1–3** and **3c** S33

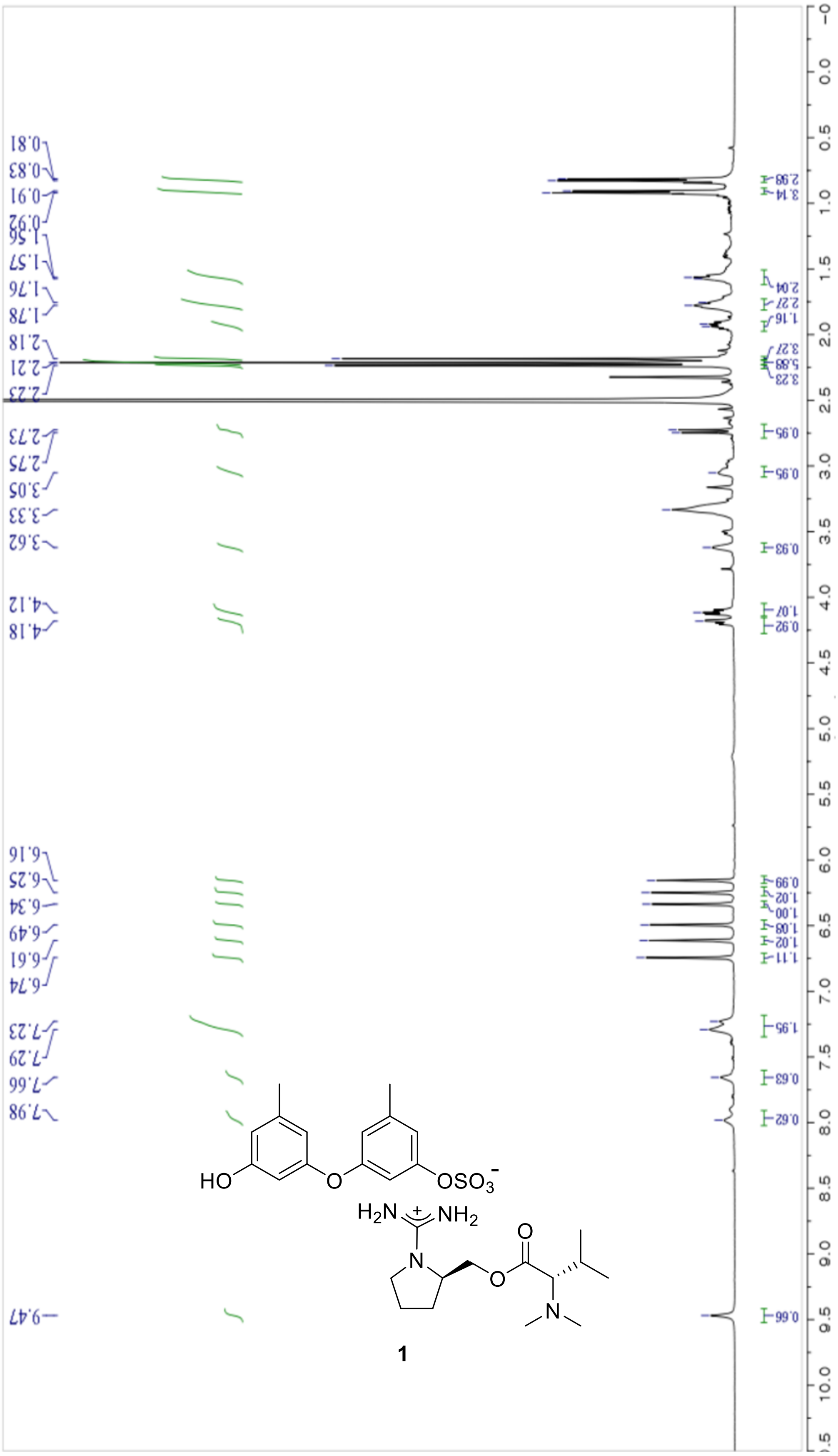

Figure S1. The ^{1}H NMR (500 MHz, DMSO-d_6) spectrum of **1**

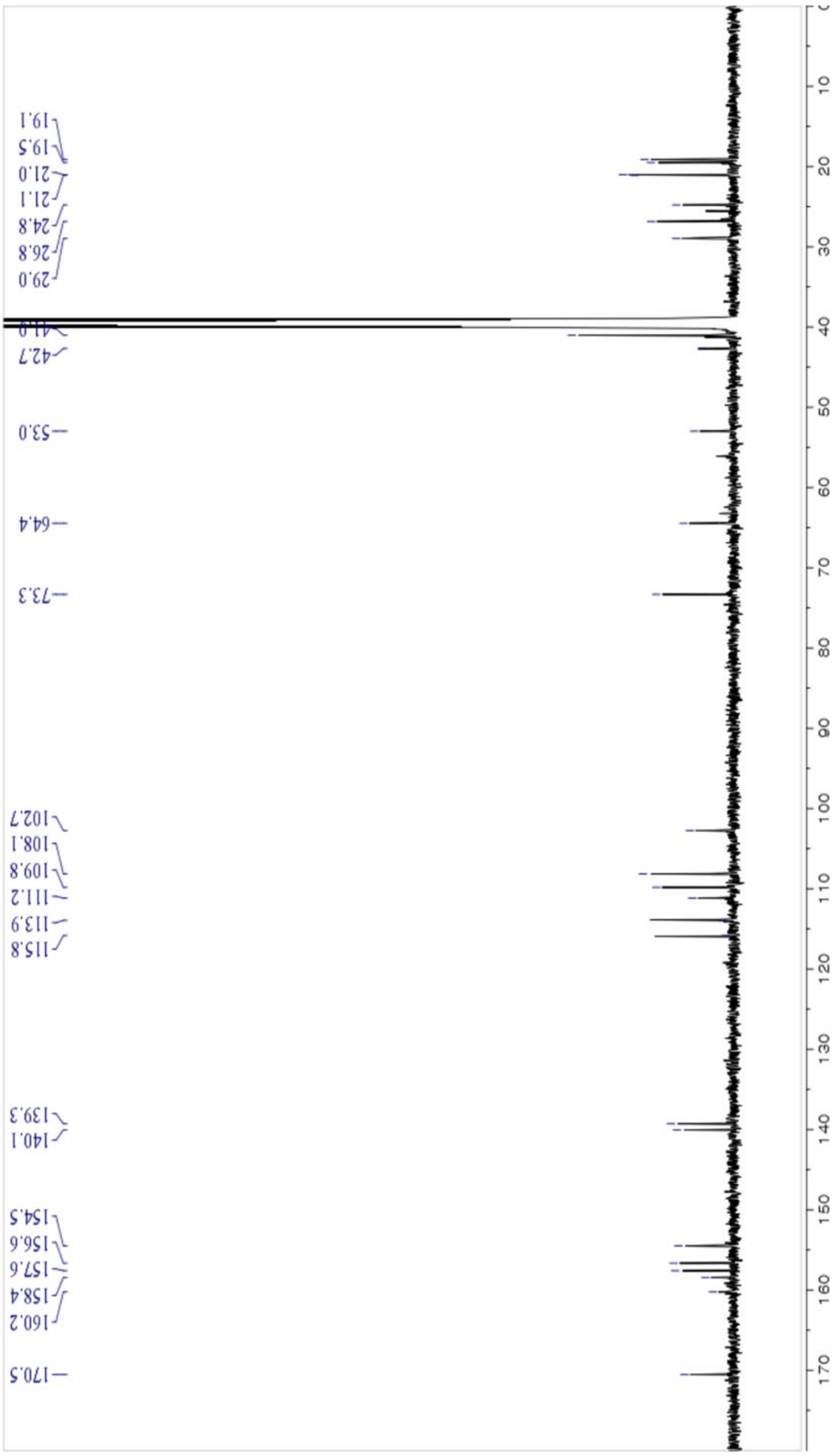

Figure S2. The ^{13}C NMR (125 MHz, DMSO-d_6) spectrum of **1**

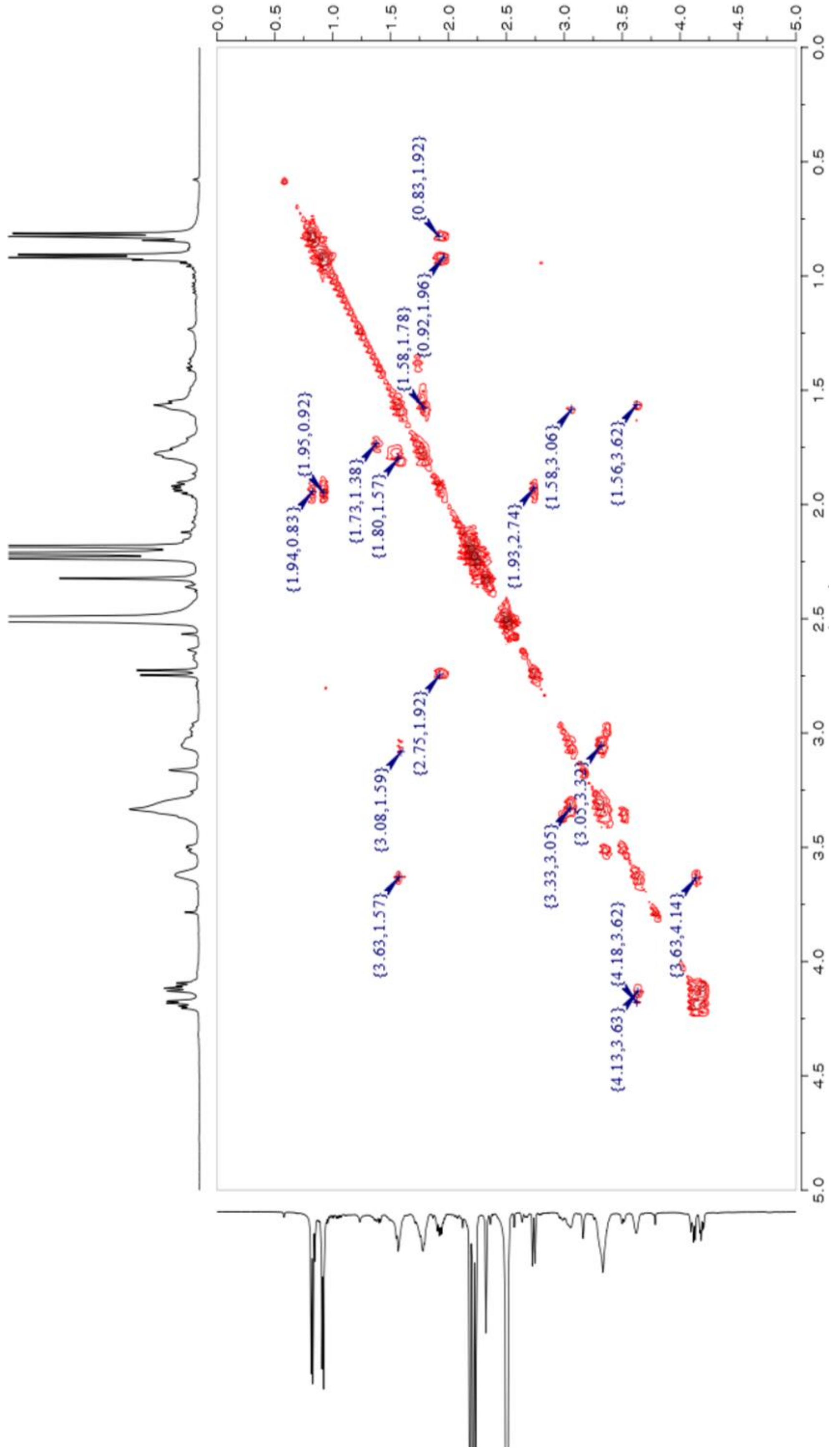

Figure S3. The COSY (500 MHz, DMSO-d_6) spectrum of **1**

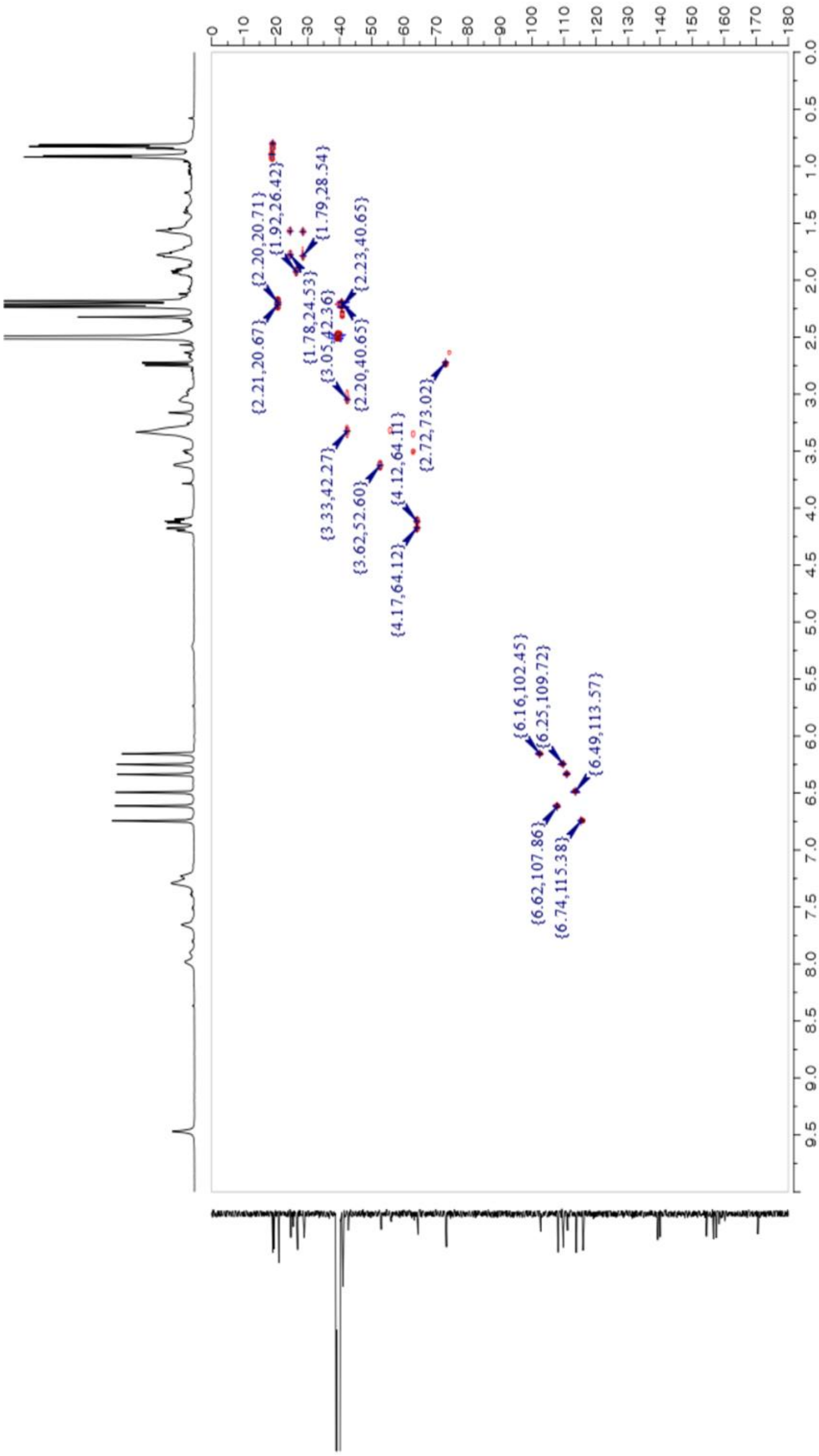

Figure S4. The HSQC (500 MHz, DMSO-d_6) spectrum of **1**

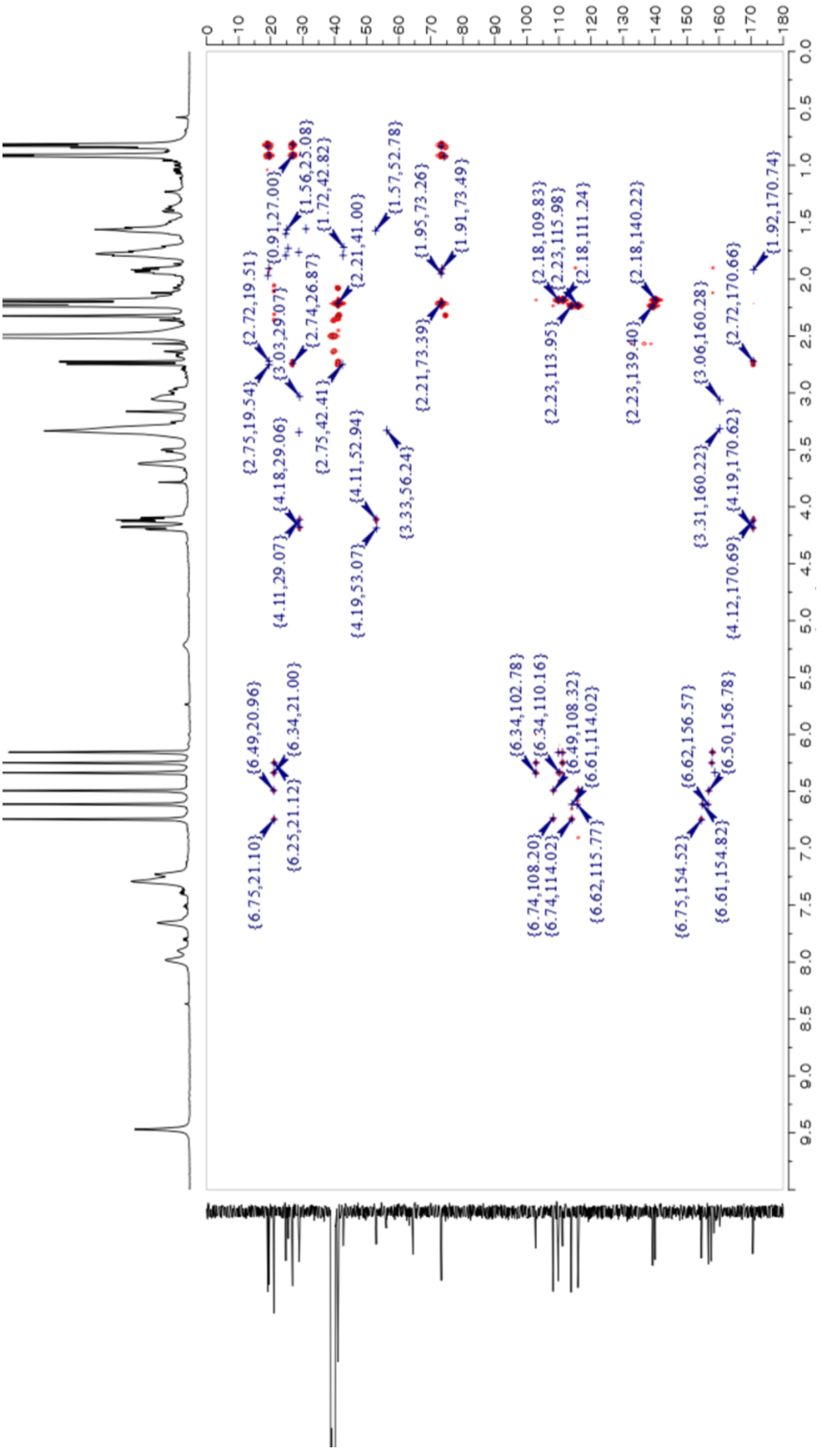

Figure S5. The HMBC (500 MHz, DMSO-d_6) spectrum of **1**

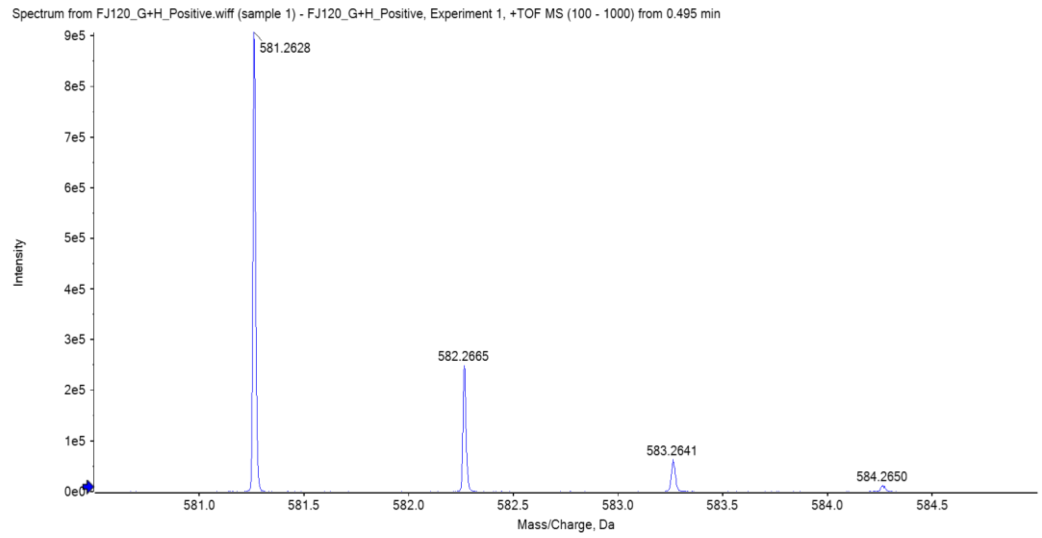

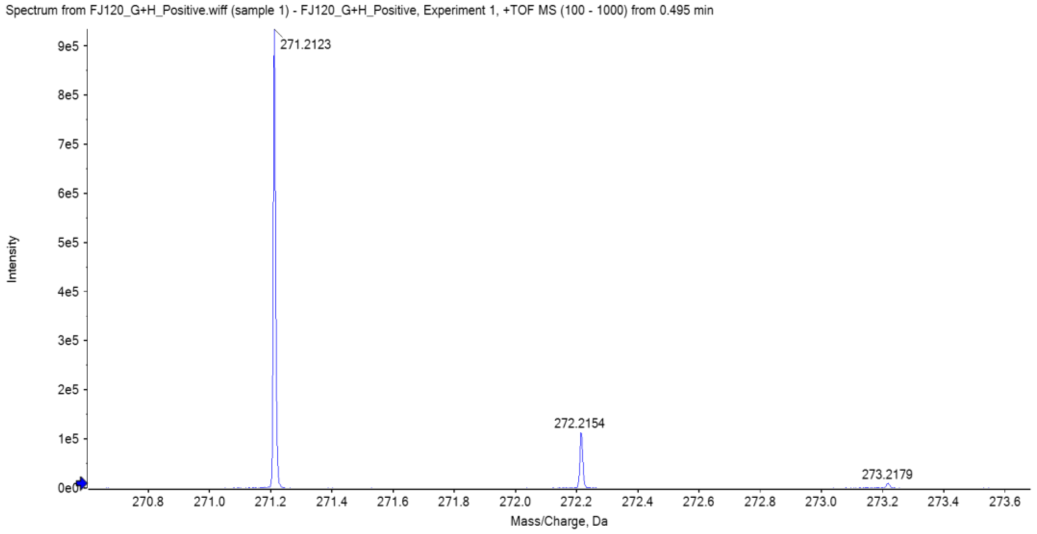

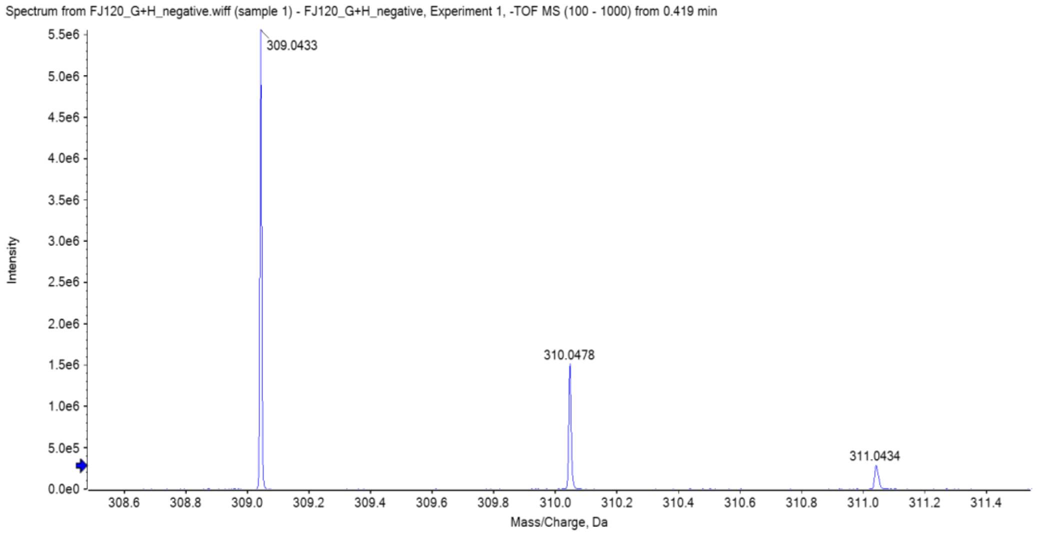

Figure S6. HR-ESI-MS data of **1**

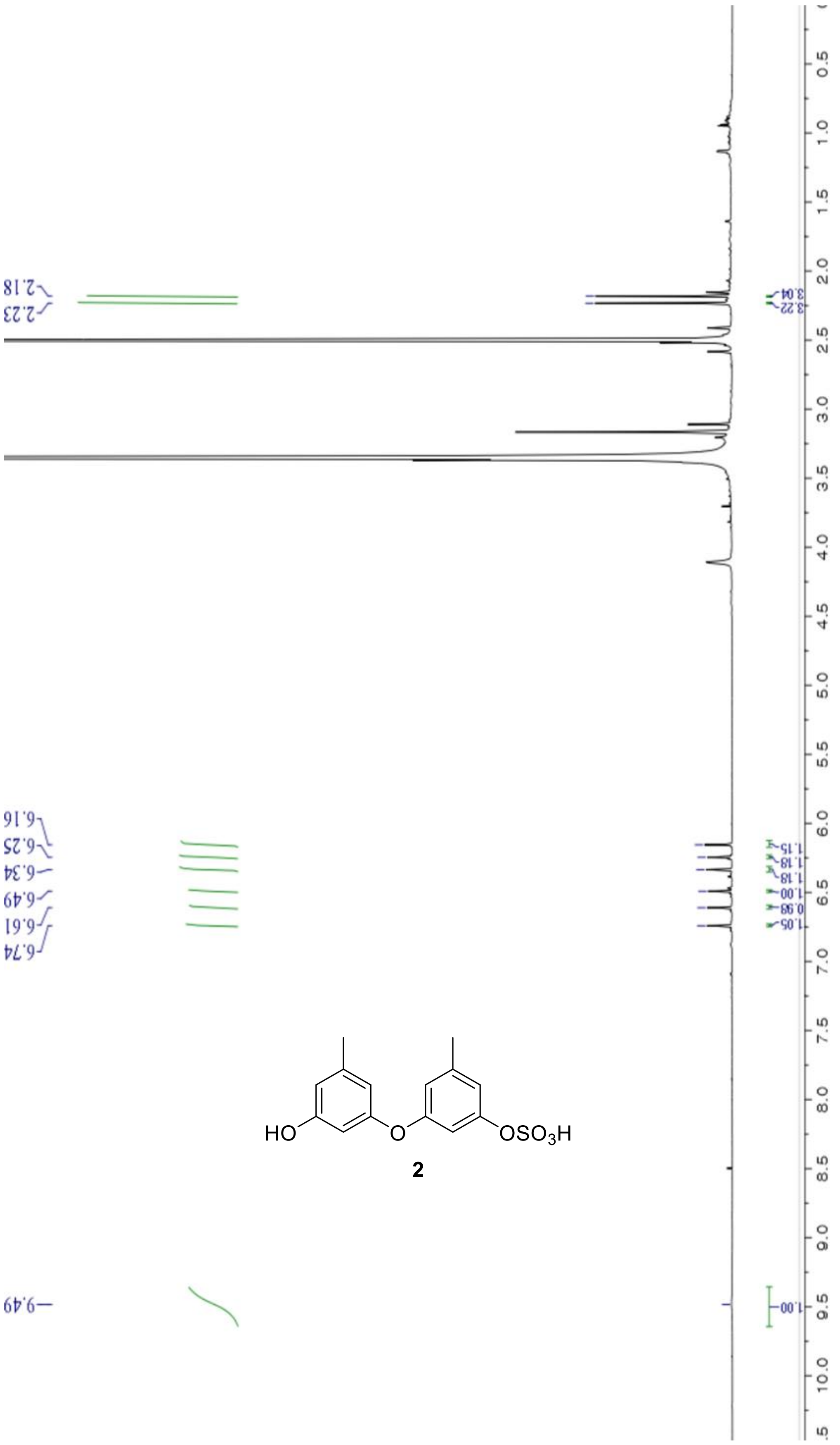

Figure S7. The ^{1}H NMR (600 MHz, DMSO- d_6) spectrum of **2**

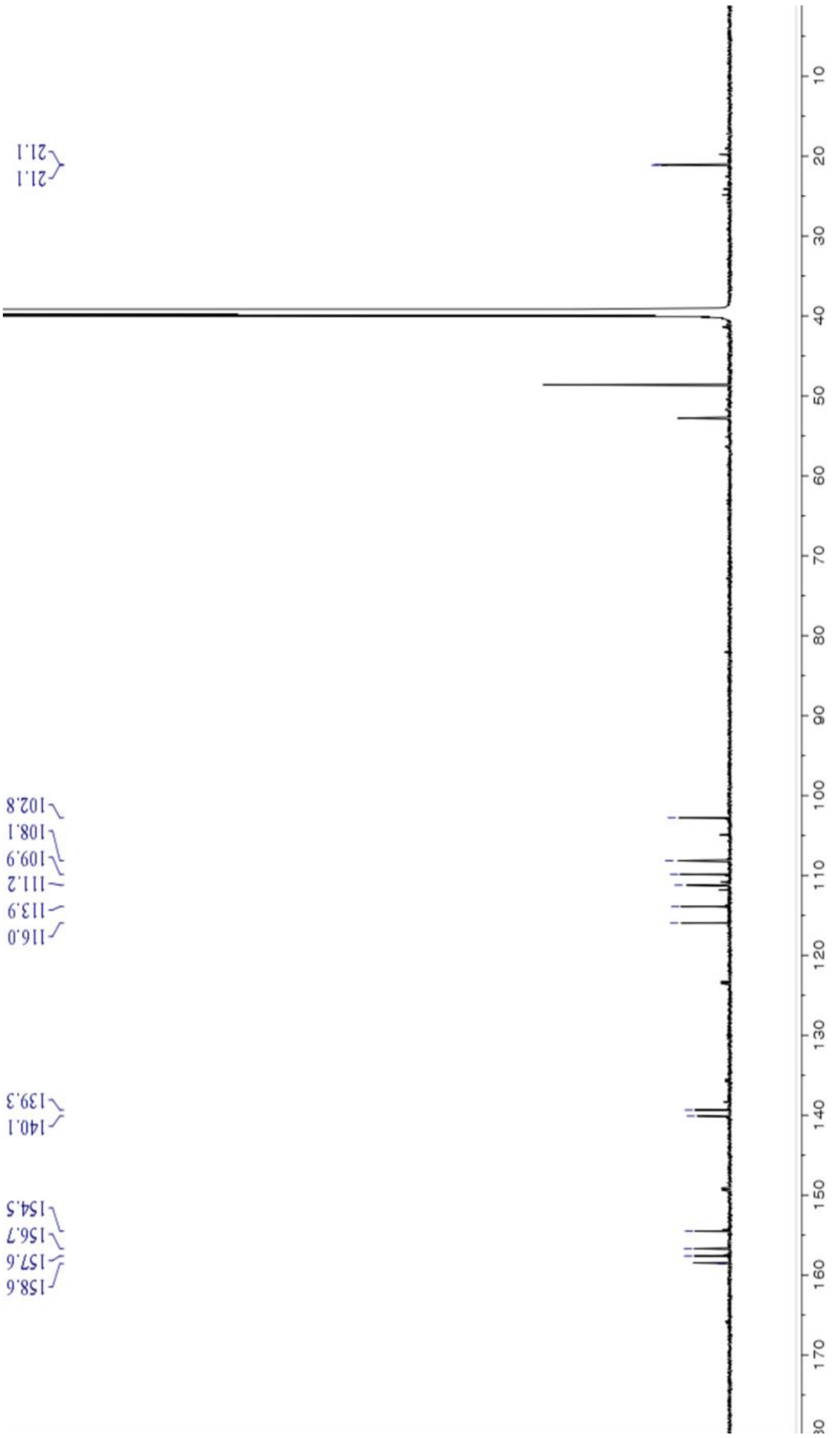

Figure S8. The ^{13}C NMR (150 MHz, DMSO- d_6) spectrum of **2**

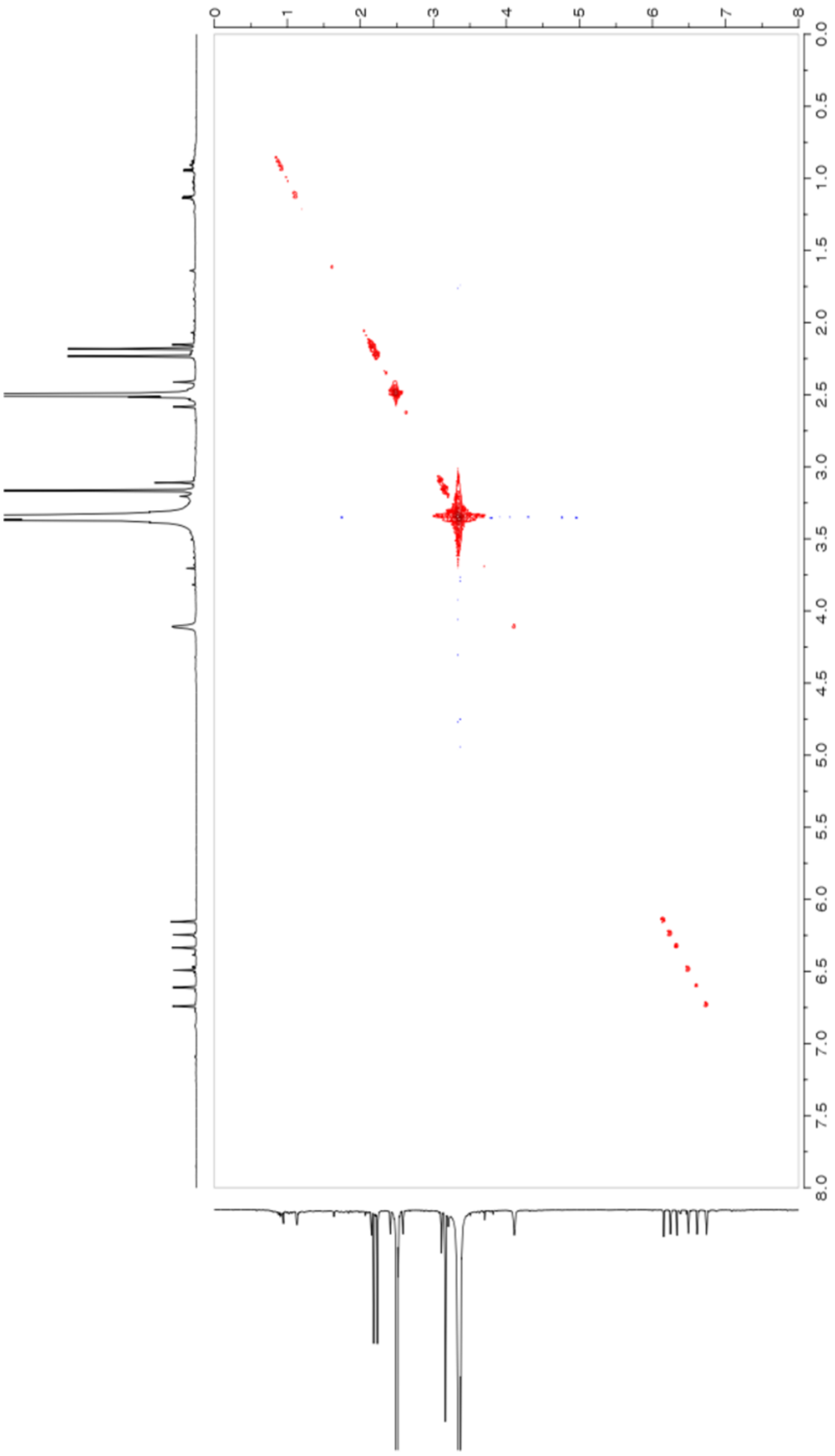

Figure S9. The COSY (600 MHz, DMSO- d_6) spectrum of **2**

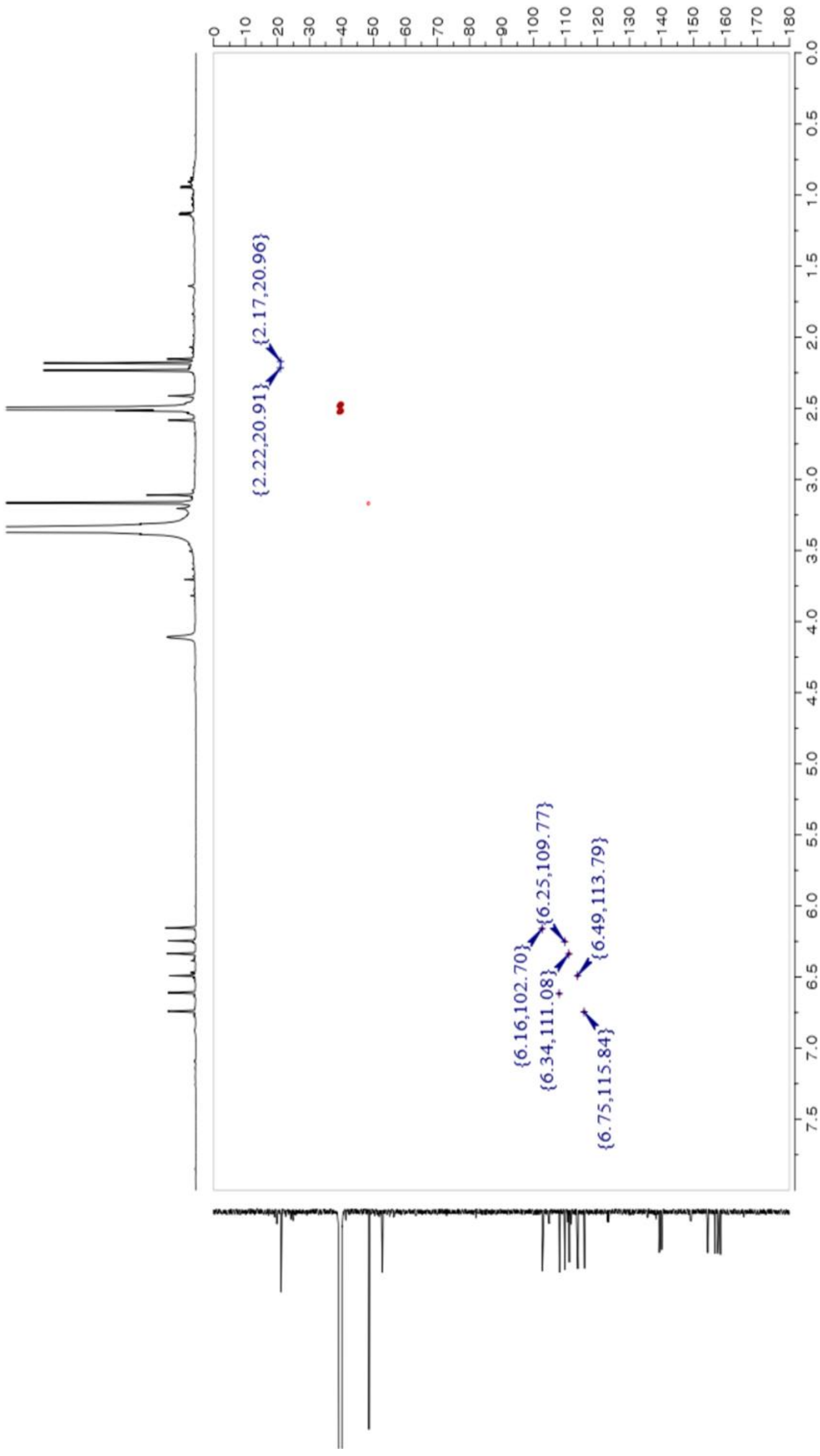

Figure S10. The HSQC (600 MHz, DMSO- d_6) spectrum of **2**

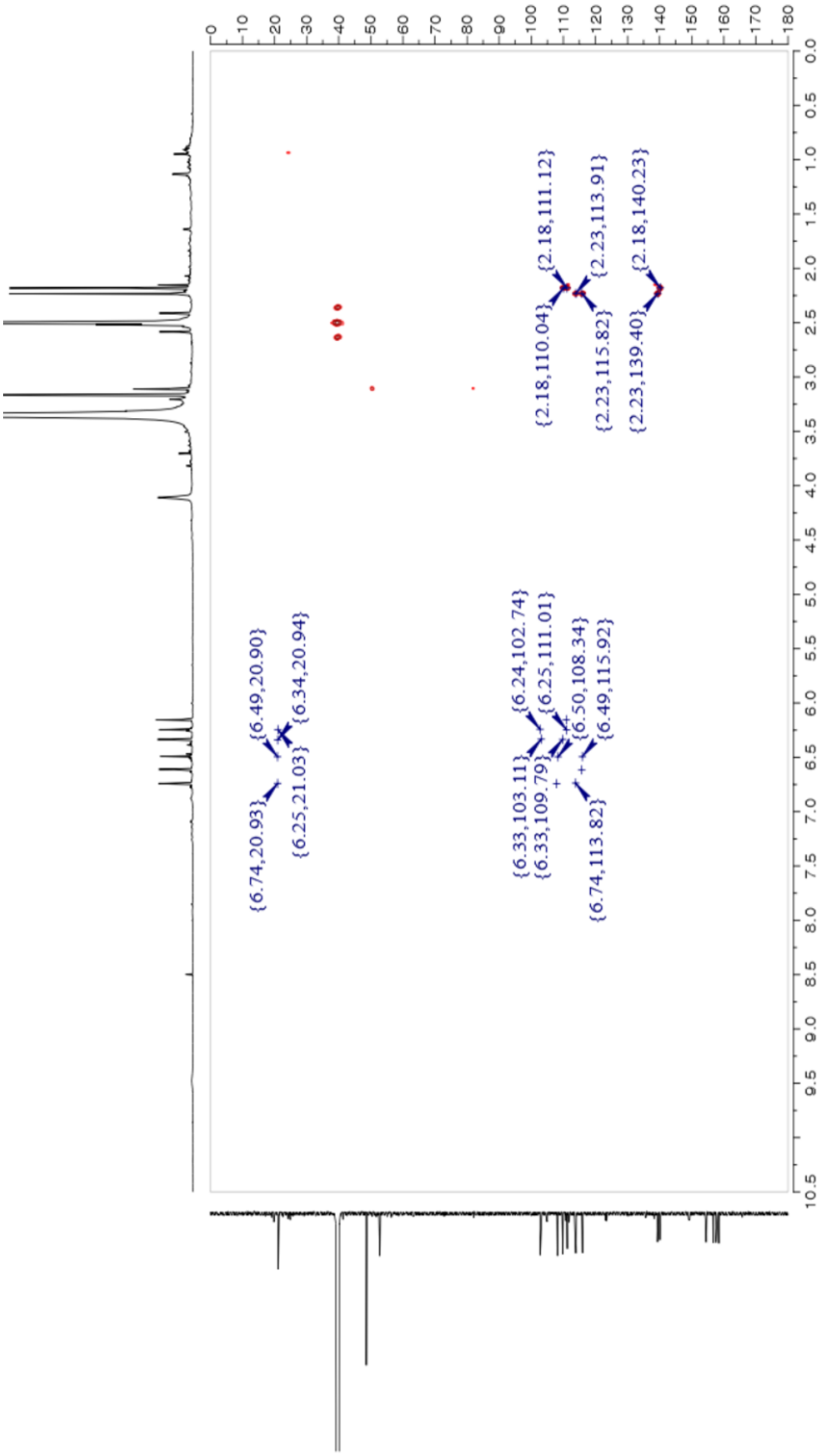

Figure S11. The HMBC (600 MHz, DMSO- d_6) spectrum of **2**

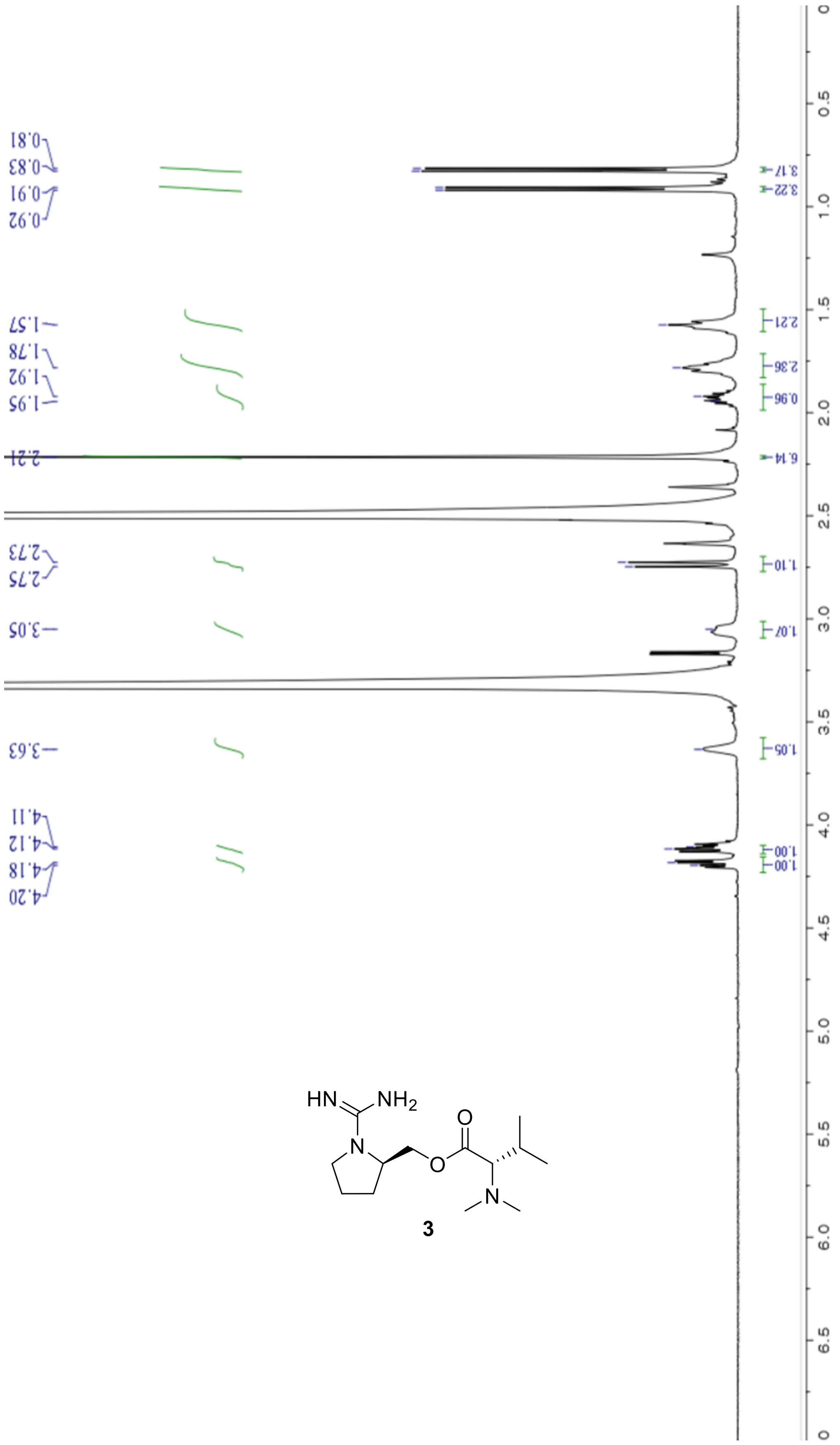

Figure S12. The ^{1}H NMR (600 MHz, DMSO-d_6) spectrum of **3**

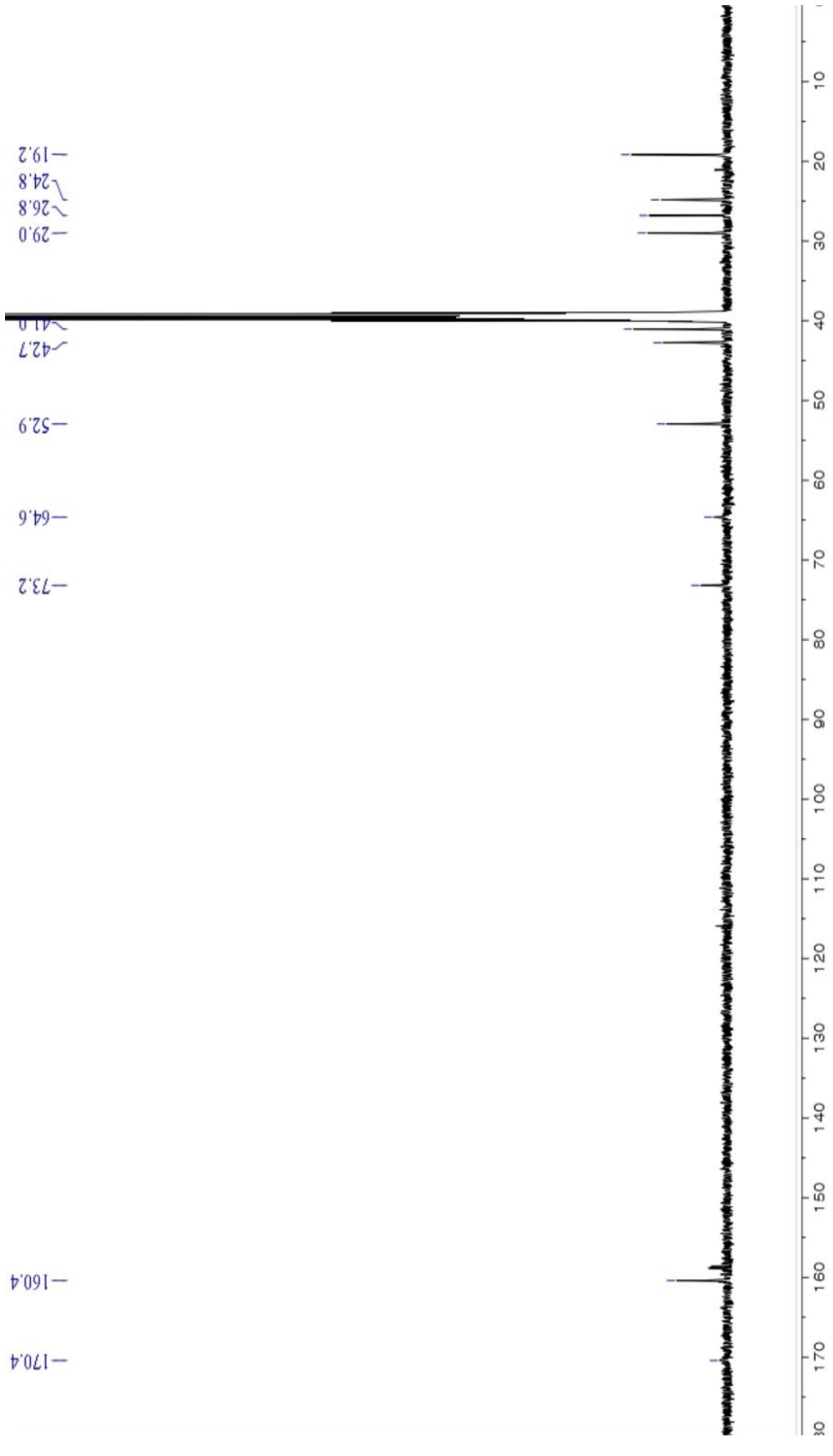

Figure S13. The ^{13}C NMR (150 MHz, DMSO-d_6) spectrum of **3**

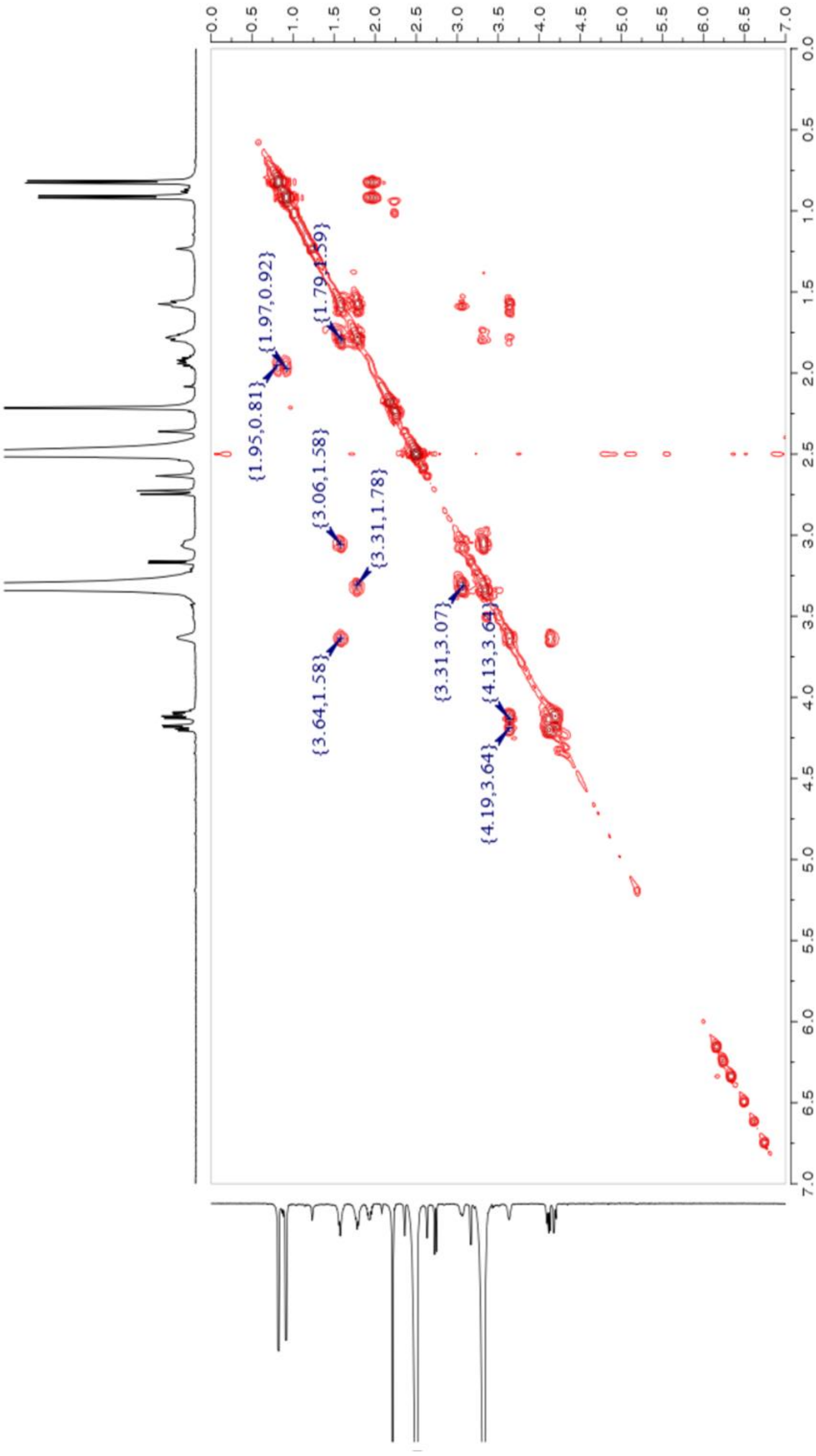

Figure S14. The COSY (600 MHz, DMSO-d_6) spectrum of **3**

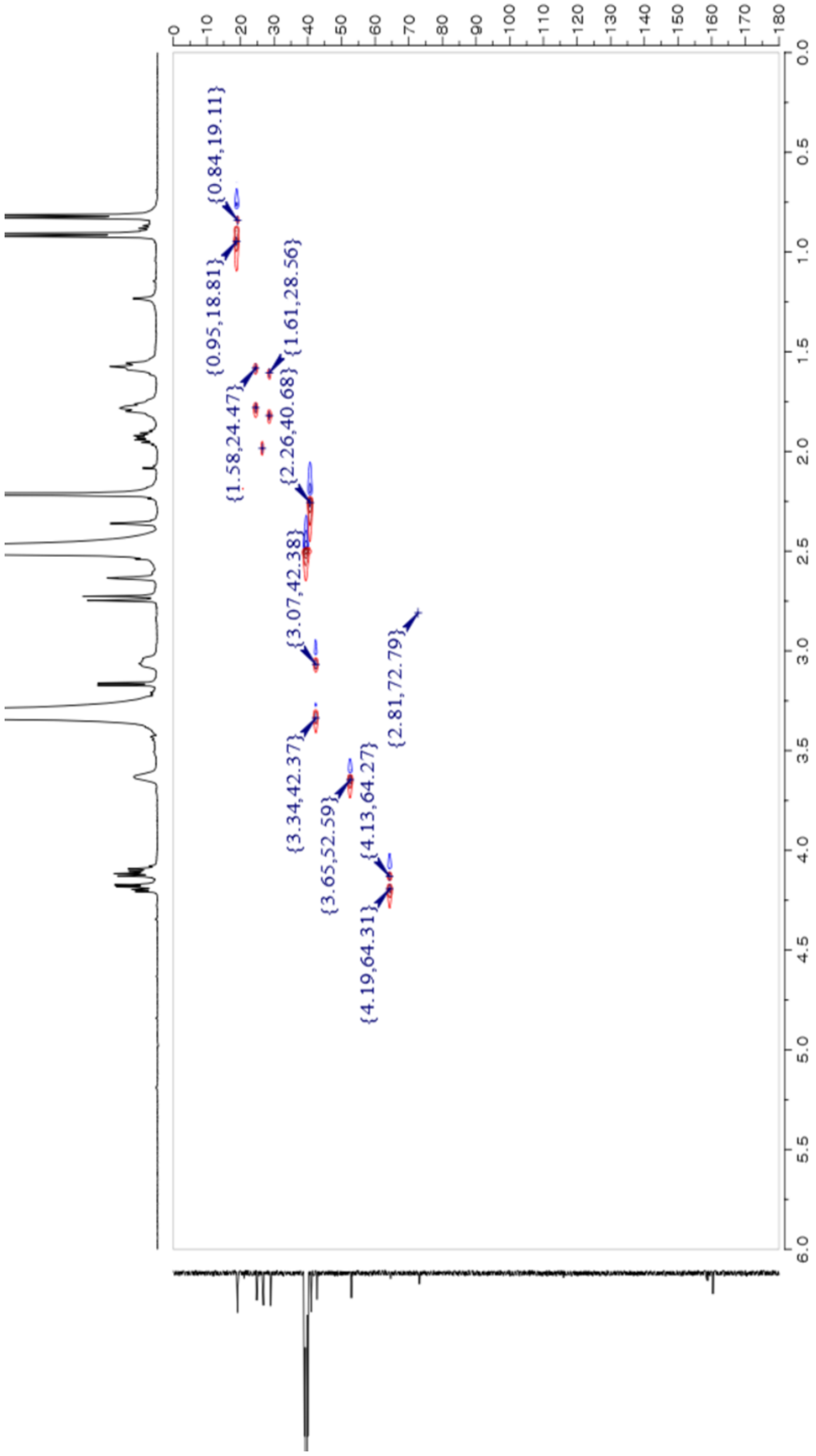

Figure S15. The HSQC (600 MHz, DMSO-d_6) spectrum of **3**

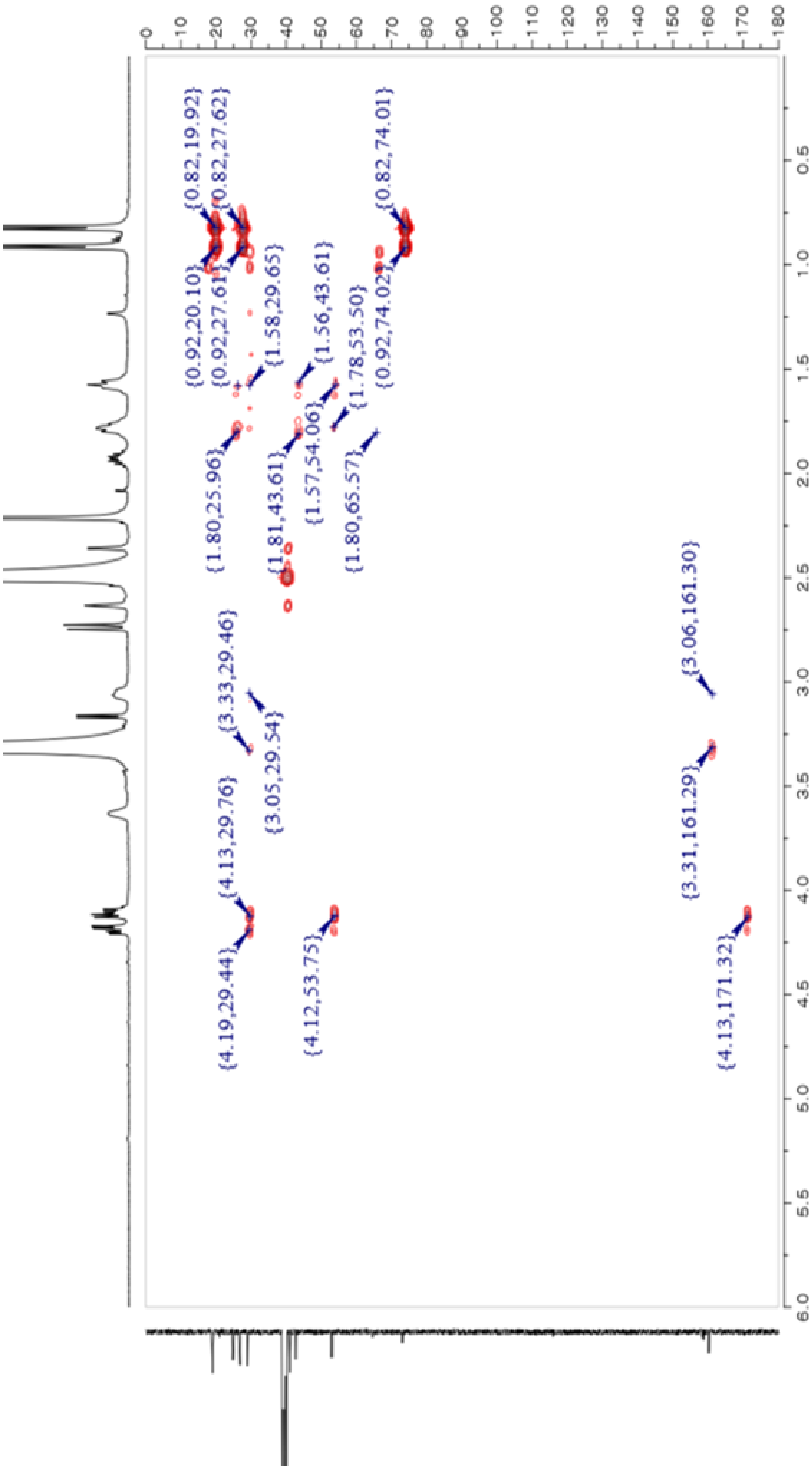

Figure S16. The HMBC (600 MHz, DMSO-d_6) spectrum of **3**

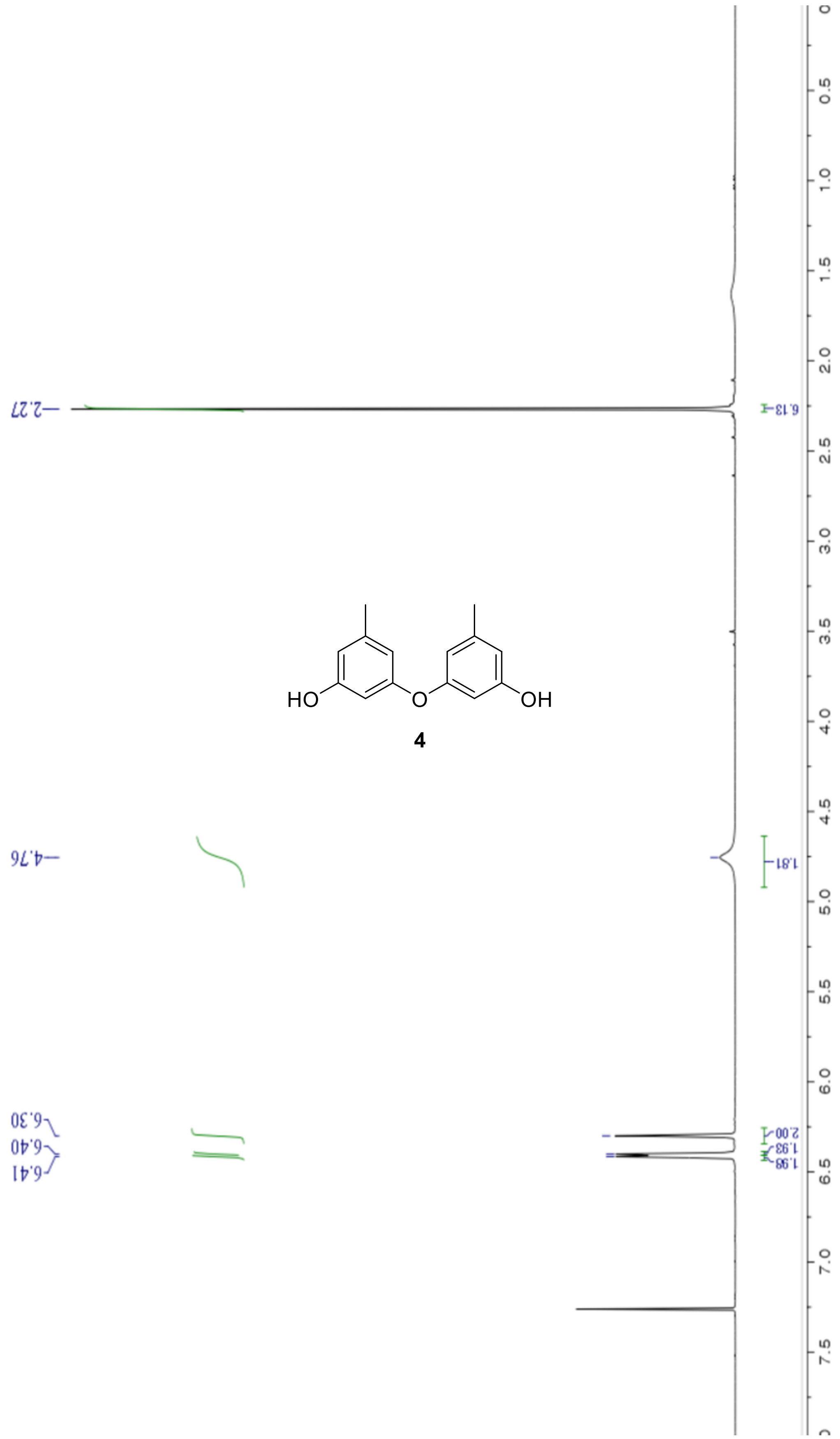

Figure S17. The ^{1}H NMR (400 MHz, $CDCl_3$) spectrum of **4**

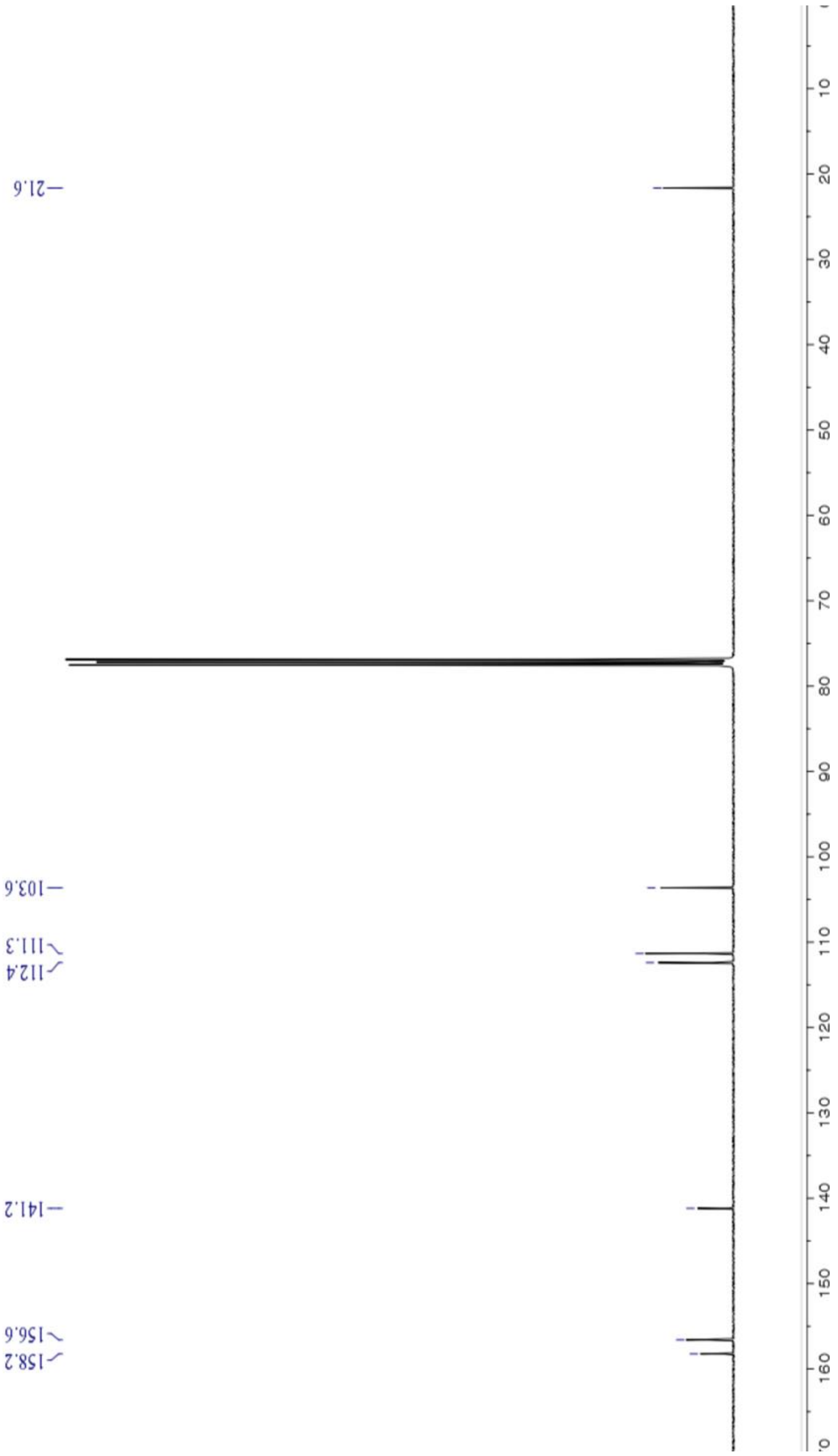

Figure S18. The ^{13}C NMR (100 MHz, $CDCl_3$) spectrum of **4**

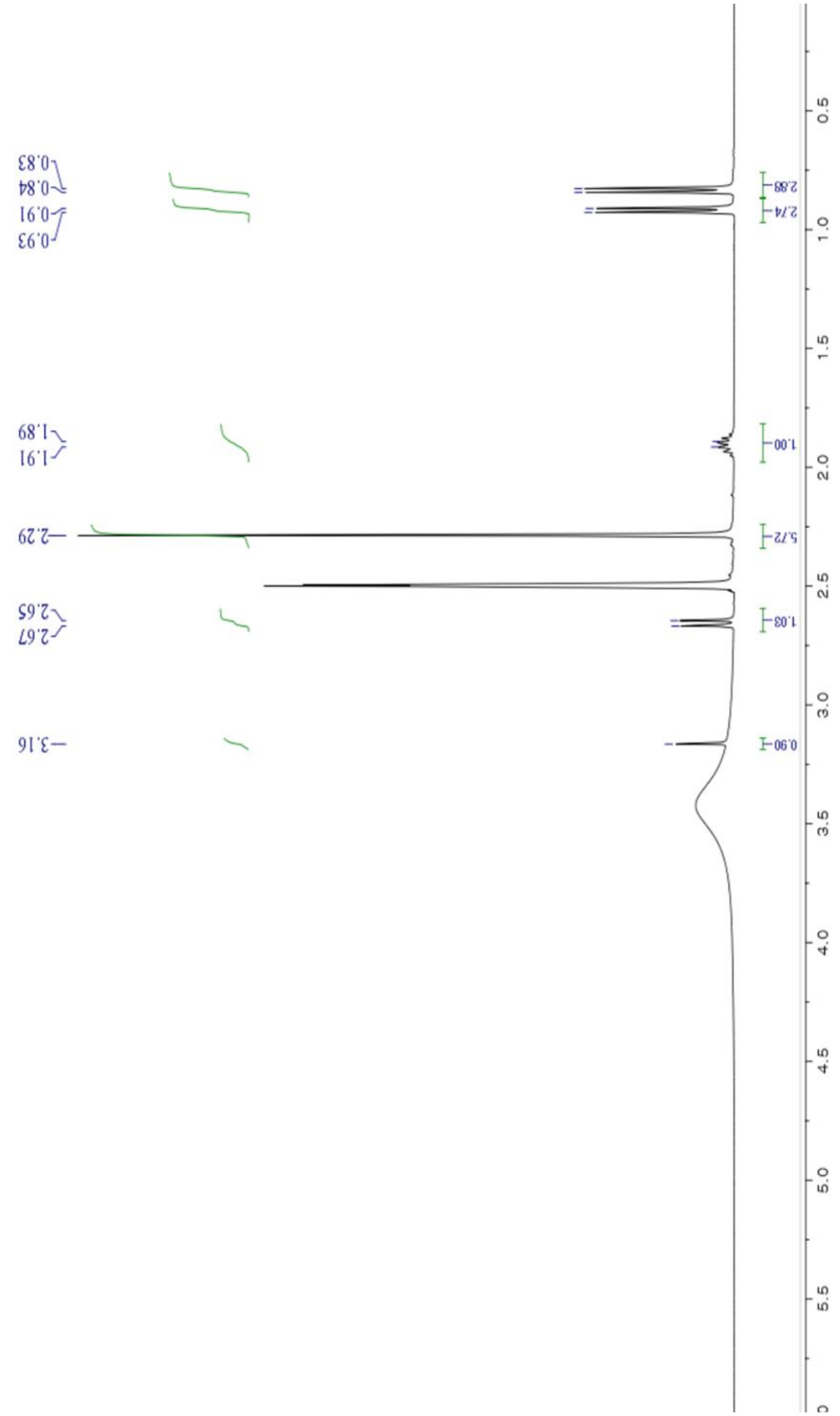

Figure S19. The ^{1}H NMR (400 MHz, DMSO-d_6) spectrum of **3a**

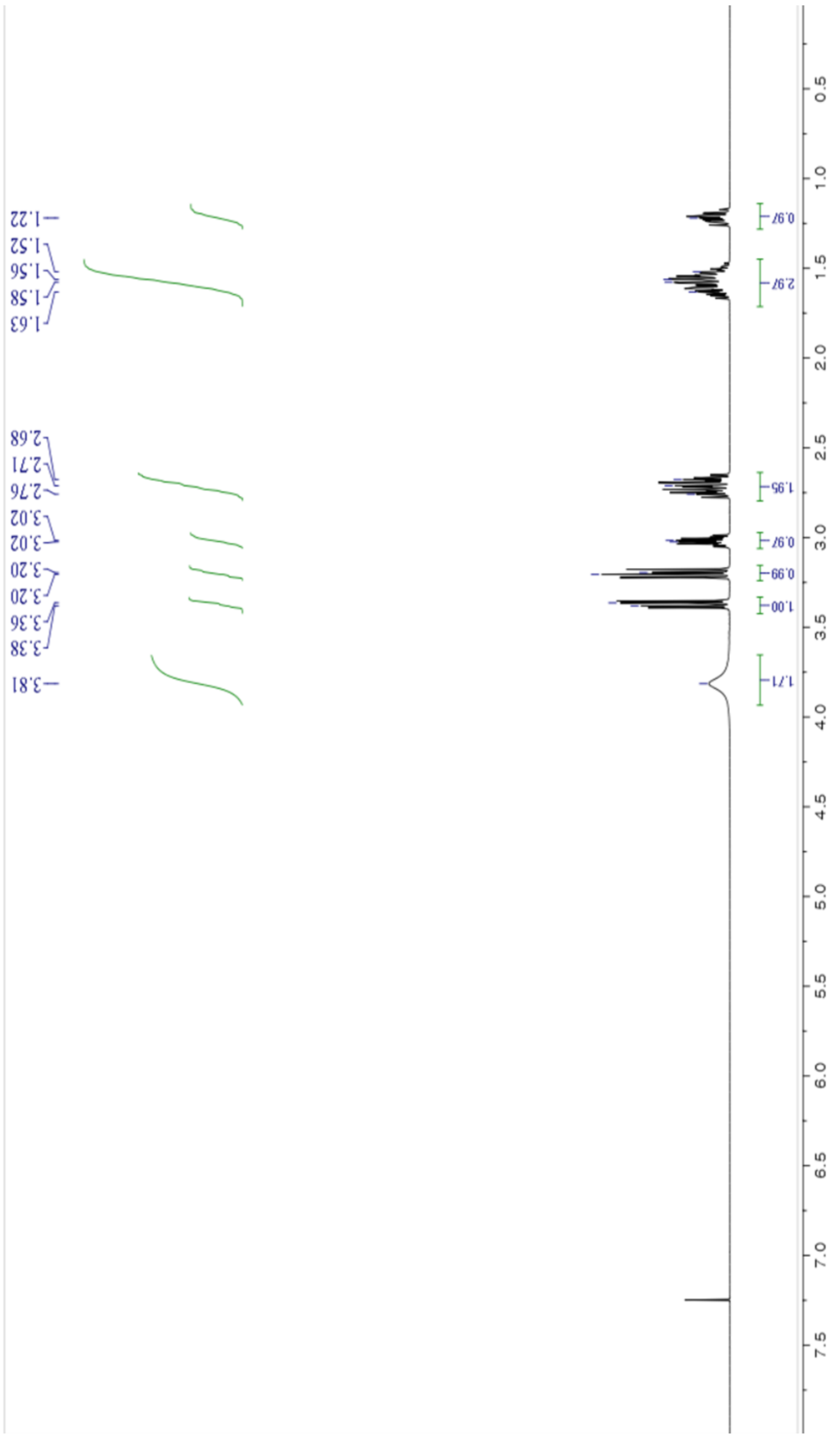

Figure S20. The ^{1}H NMR (400 MHz, $CDCl_3$) spectrum of **3b**

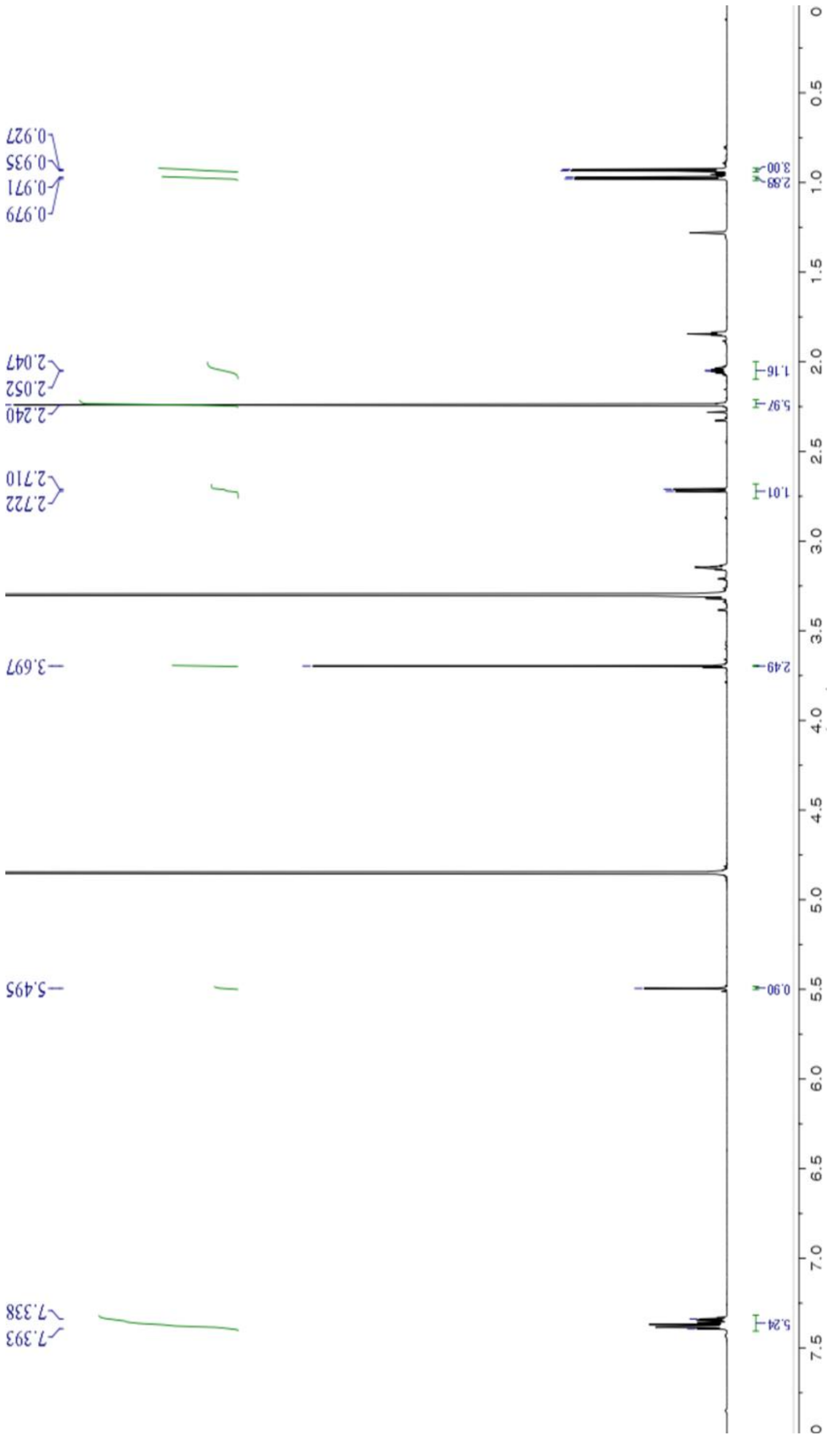

Figure S21. The ^{1}H NMR (800 MHz, Methanol-d_4) spectrum of **3a*S***

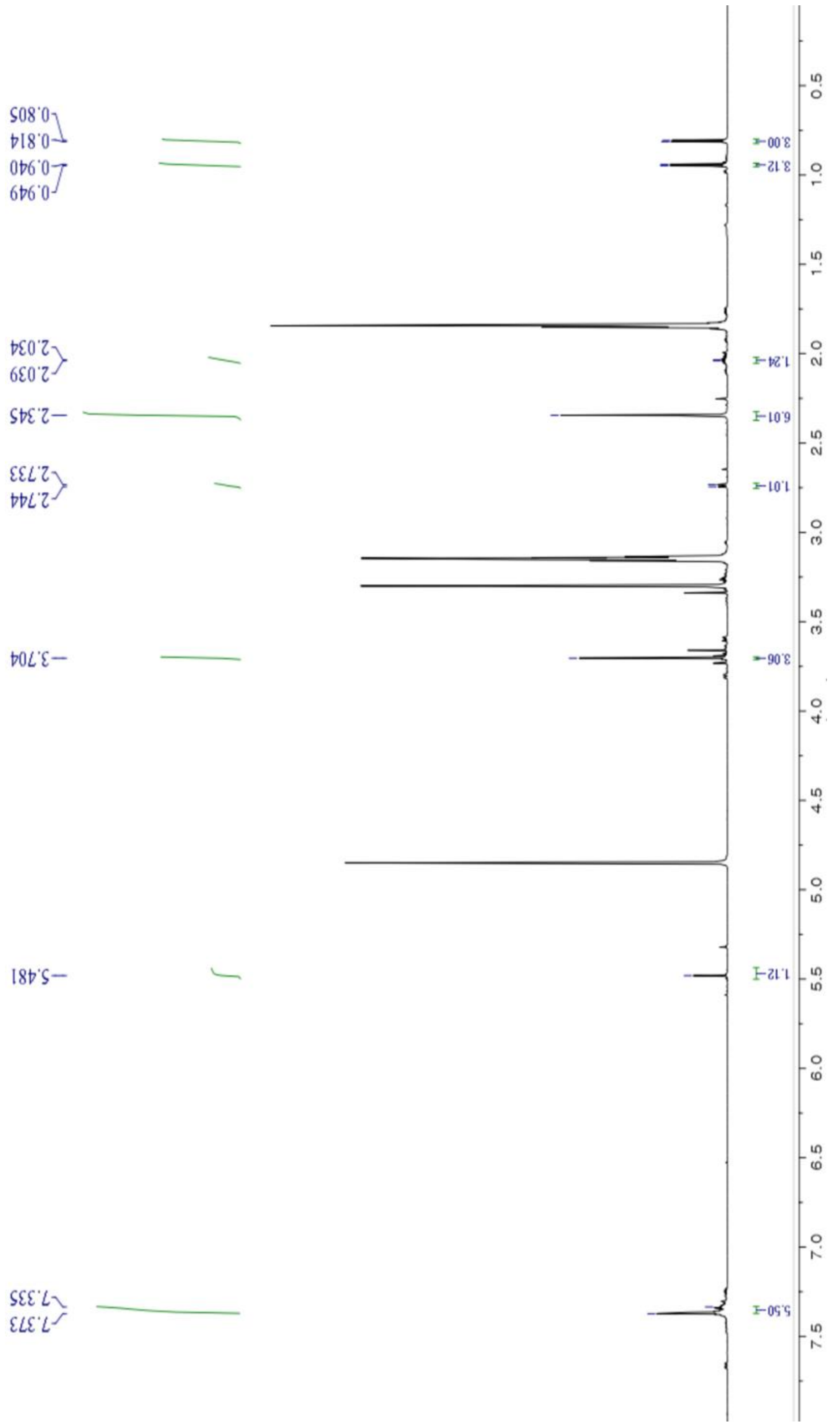

Figure S22. The ^{1}H NMR (800 MHz, Methanol-d_4) spectrum of **3a*R***

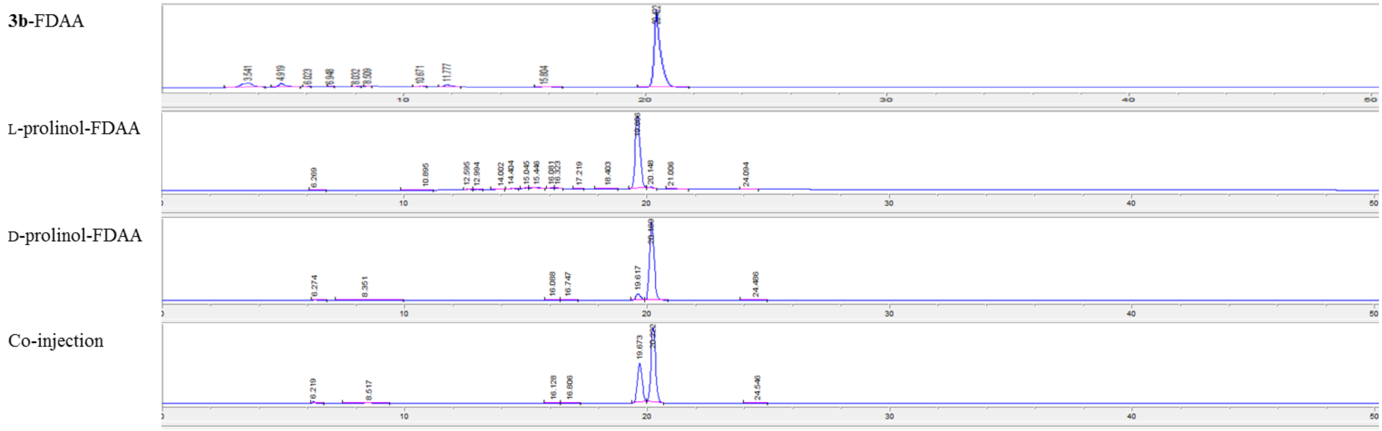

Figure S23. The LC/MS chromatogram data of **3b-L-FDAA** adduct

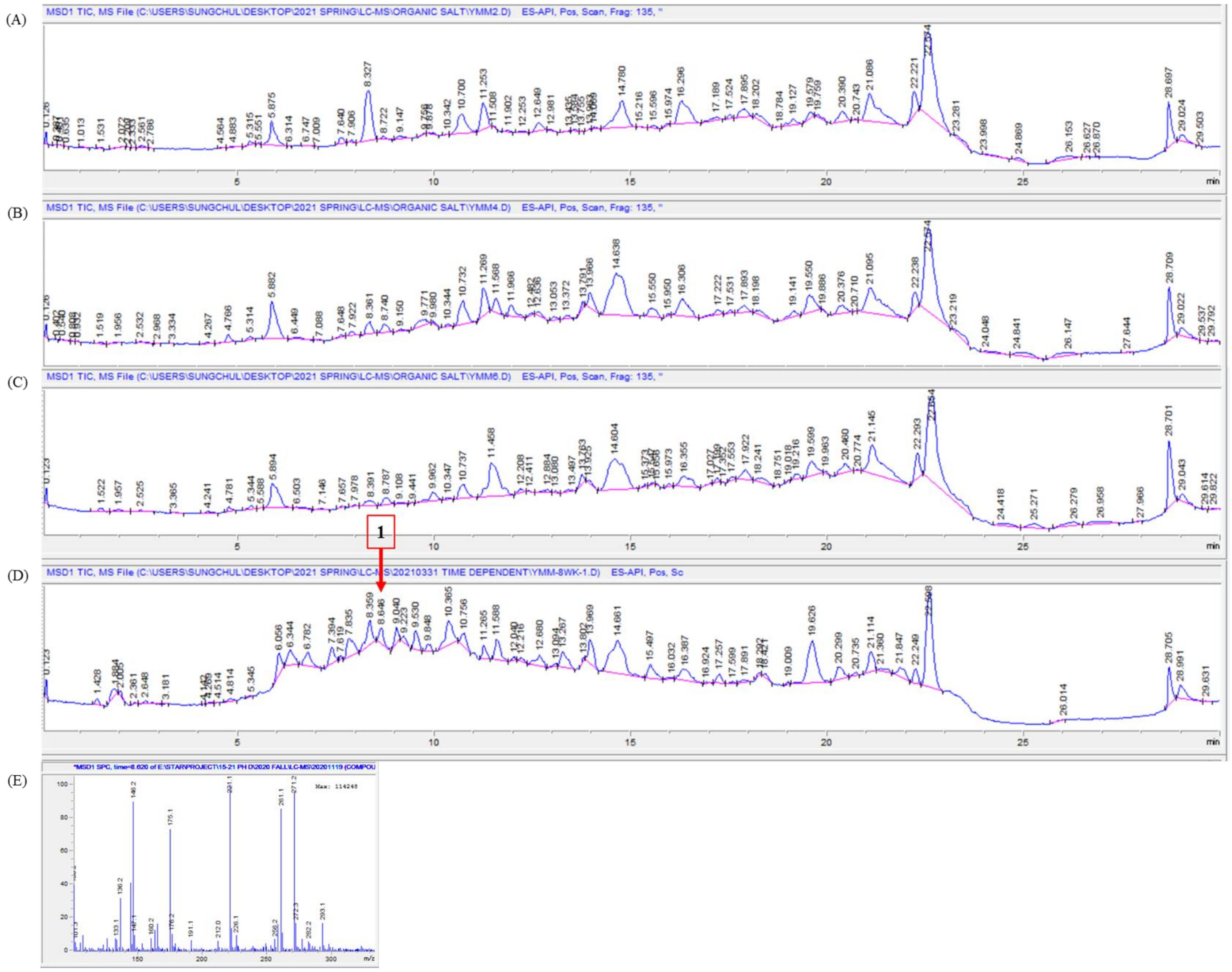

Figure S24. The time-scale LC-MS analysis of YMM liquid media extracts. (A)–(D) Extracts from 2, 4, 6, and 8 weeks of incubation, respectively. (E) The MS data on t_R = 8.641. *Condition*: YMC-ODS-A column, 250 x 4.6 mm; 10-100% MeCN-H_2O gradient over 20 min with 0.1% trifluoroacetic acid. Compound **1** (t_R = 8.646).

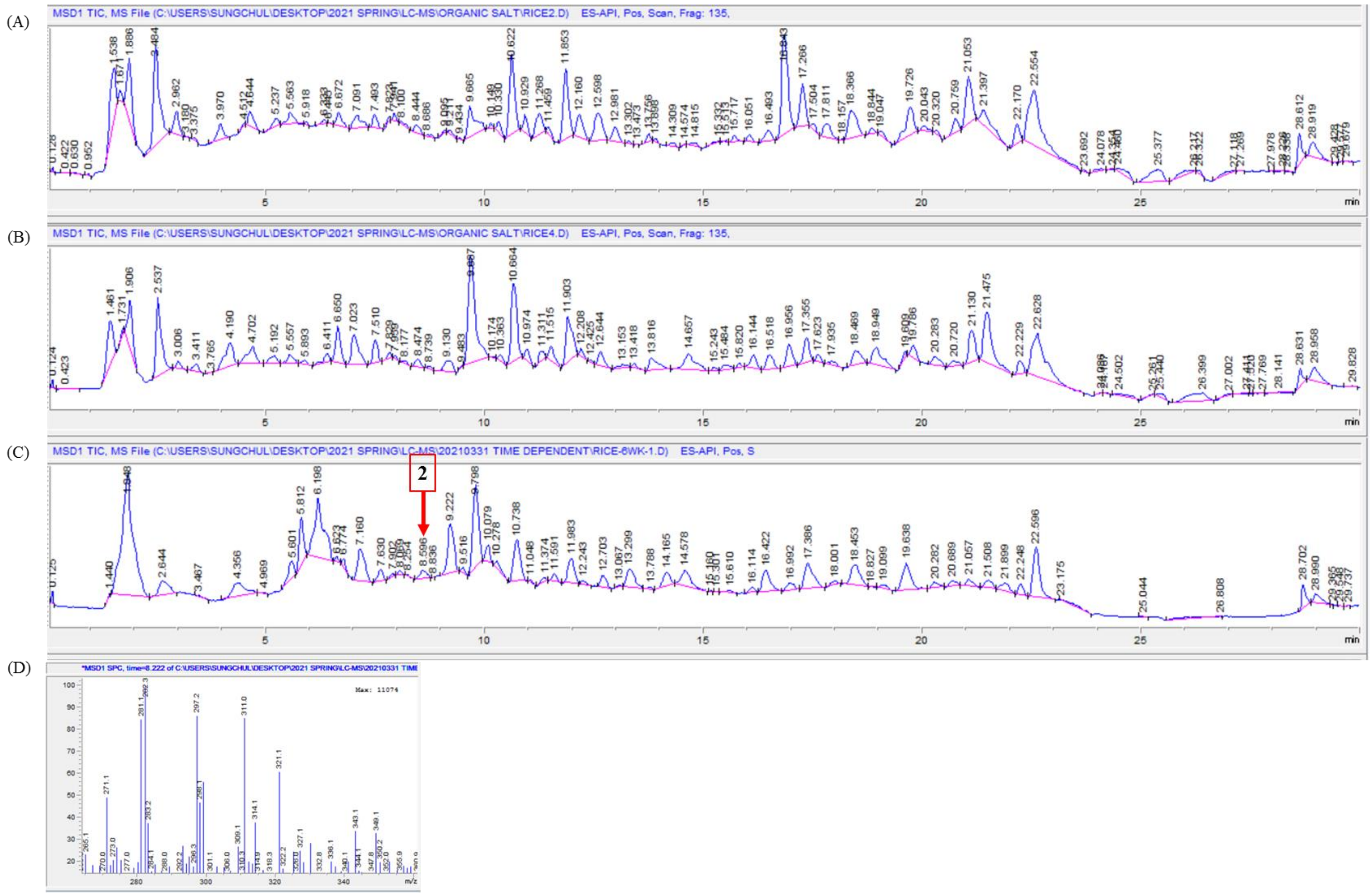

Figure S25. The time-scale LC-MS analysis of YMM-rice semi-solid media extracts. (A)–(C) Extracts from 2, 4 and 6 weeks of incubation, respectively. (D) The MS data on t_R = 8.596. *Condition*: YMC-ODS-A column, 250 x 4.6 mm; 10-100% MeCN-H_2O gradient over 20 min with 0.1% trifluoroacetic acid. Compound **2** (t_R = 8.596).

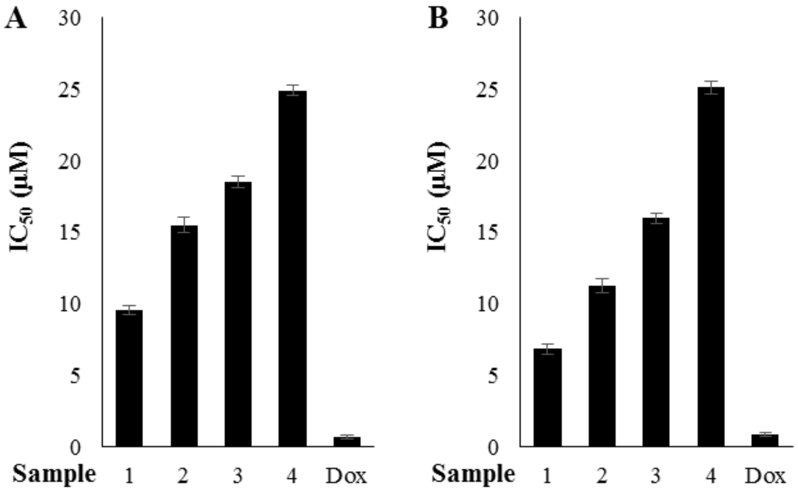

Figure S26. The MTT assay against K562 and A549 cancer cells. (A) IC_{50} values were calculated by MTT assay results against K562 cancer cell. (B) IC_{50} values were calculated by MTT assay results against A549 cancer cell. Doxorubicin was used as a positive control. The values are expressed as the mean ± SD of triplicate tests.

Table S1. Results of cytotoxicity tests

	IC_{50} (µM)	
Compounds	K562	A549
**1**	9.5	6.8
**2**	16	11
**3**	19	16
**4**	25	25
**Doxorubicin**	0.72	0.90

(A)

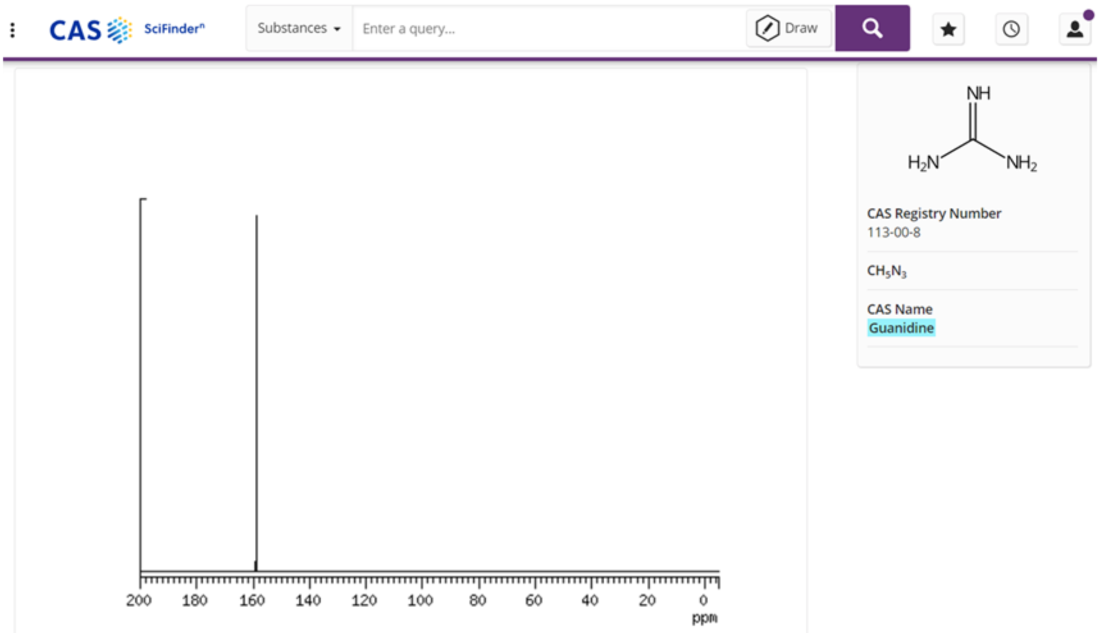

(B)

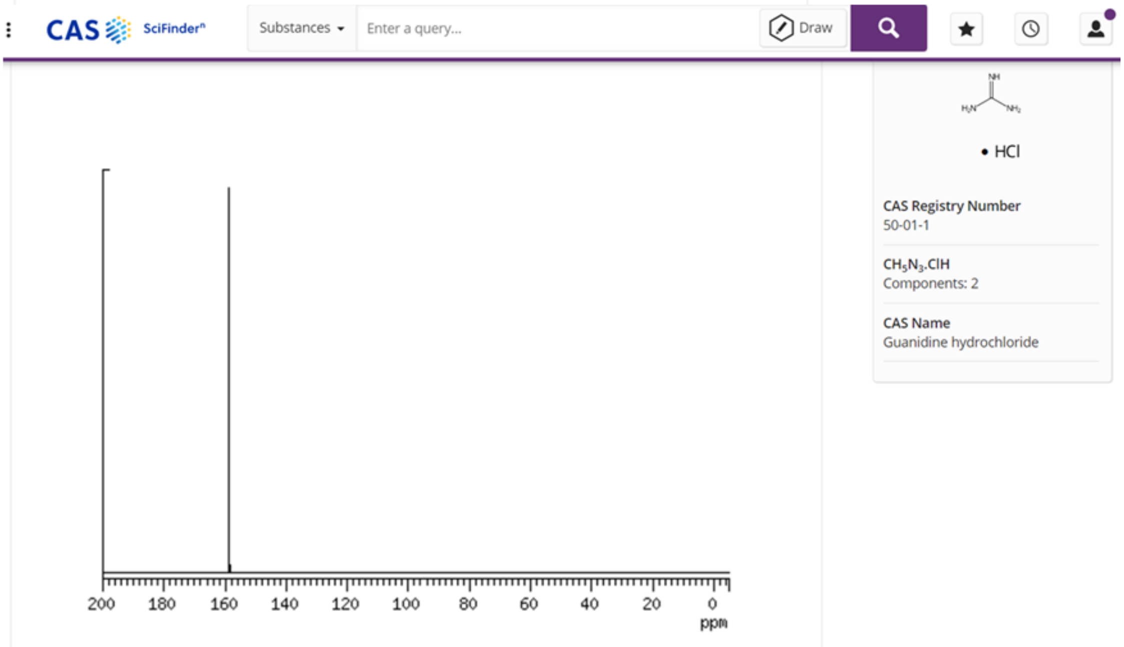

Figure S27. The ^{13}C NMR (25 MHz, D_2O) spectra of guanidine and guanidine hydrochloride. (A) The ^{13}C NMR (25 MHz, D_2O) spectrum of guanidine (CAS 113-00-8). The ^{13}C NMR (25 MHz, D_2O) spectrum of guanidine hydrochloride (CAS 50-01-1). The Scifinder provides experimental carbon NMR spectra of both guanidine (CAS 113-00-8) and guanidine hydrochloride (CAS 50-01-1) obtained from the National Institute of Advanced Industrial Science and Technology (Japan).

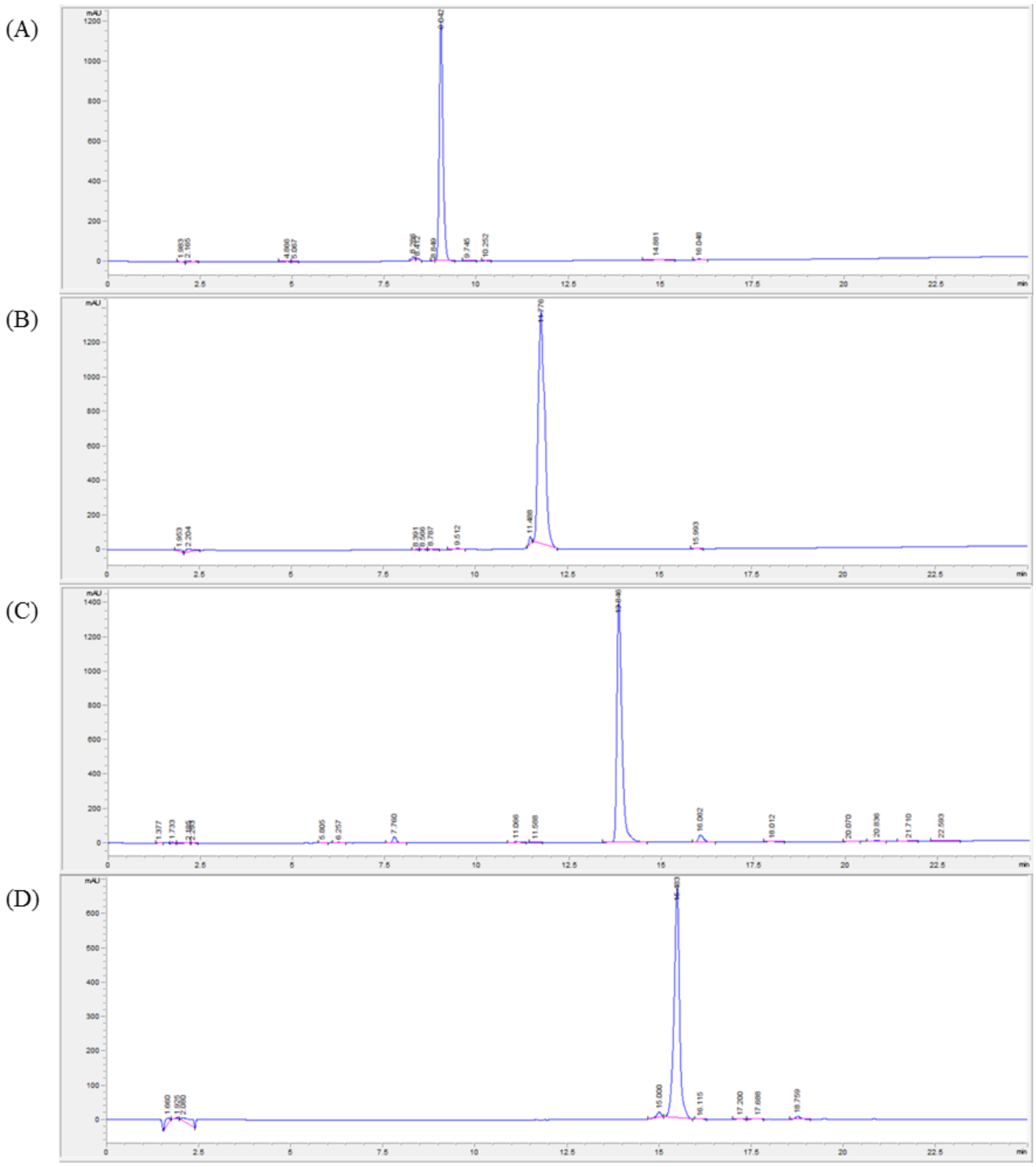

Figure S28. The HPLC analysis of **1**. *Condition*: (A) YMC-ODS-A column, 250 x 4.6 mm; 20–100% MeCN–H_2O gradient over 25 min; 0.5 mL/min; UV detection at 210 nm; t_R = 9.042 min. (B) YMC-ODS-A column, 250 x 4.6 mm; 10–100% MeCN–H_2O gradient over 25 min; 0.5 mL/min; UV detection at 210 nm; t_R = 11.776 min. (C) YMC-ODS-A column, 250 x 4.6 mm; H_2O–MeOH, 60:40; 0.5 mL/min; UV detection at 210 nm; t_R = 13.846 min. (D) YMC-ODS-A column, 250 x 4.6 mm; H_2O–MeCN, 69:31; 0.5 mL/min; UV detection at 210 nm; t_R = 15.483 min.

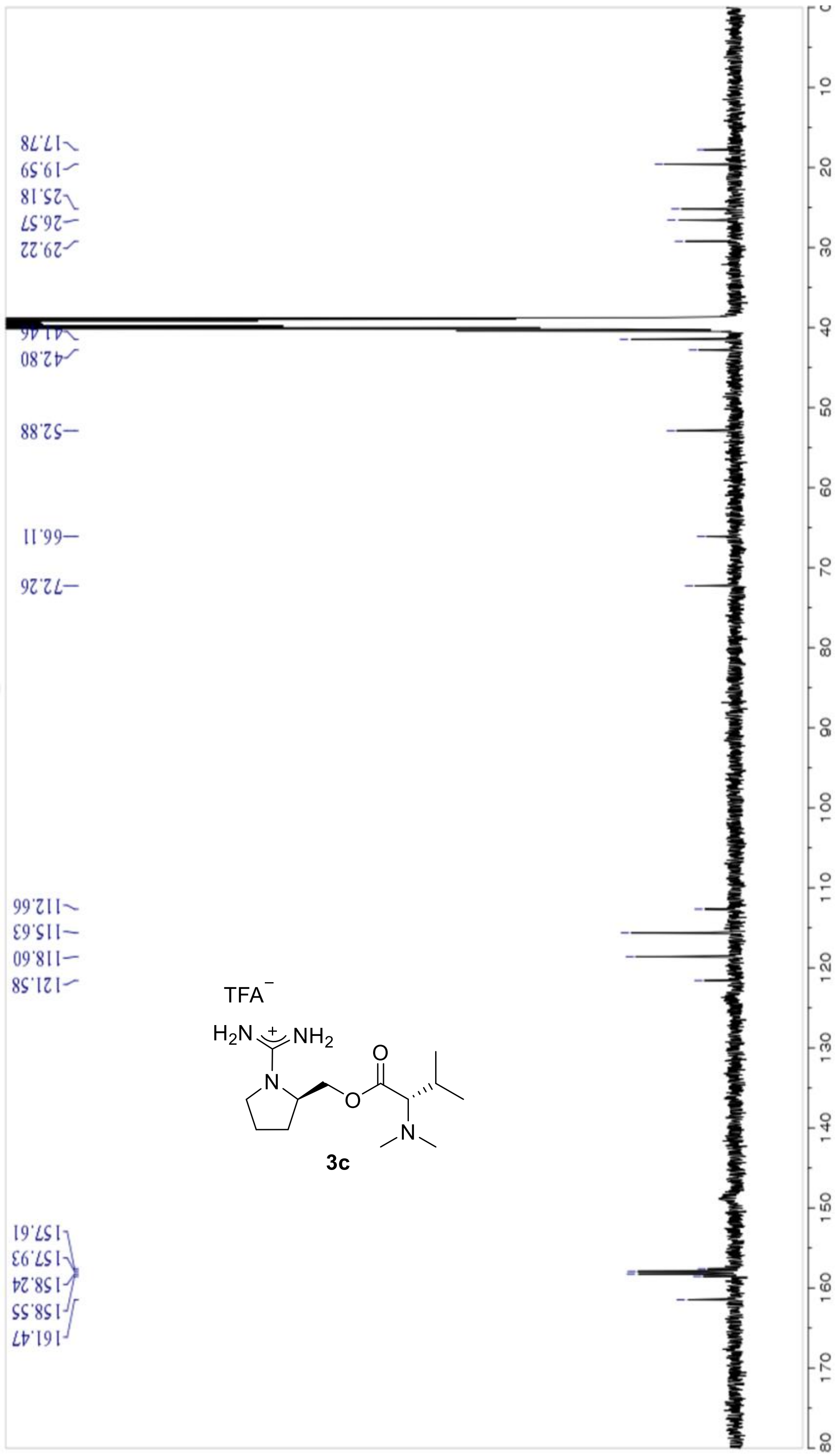

Figure S29. The ^{13}C NMR (100 MHz, DMSO-d_6) spectrum of **3c**

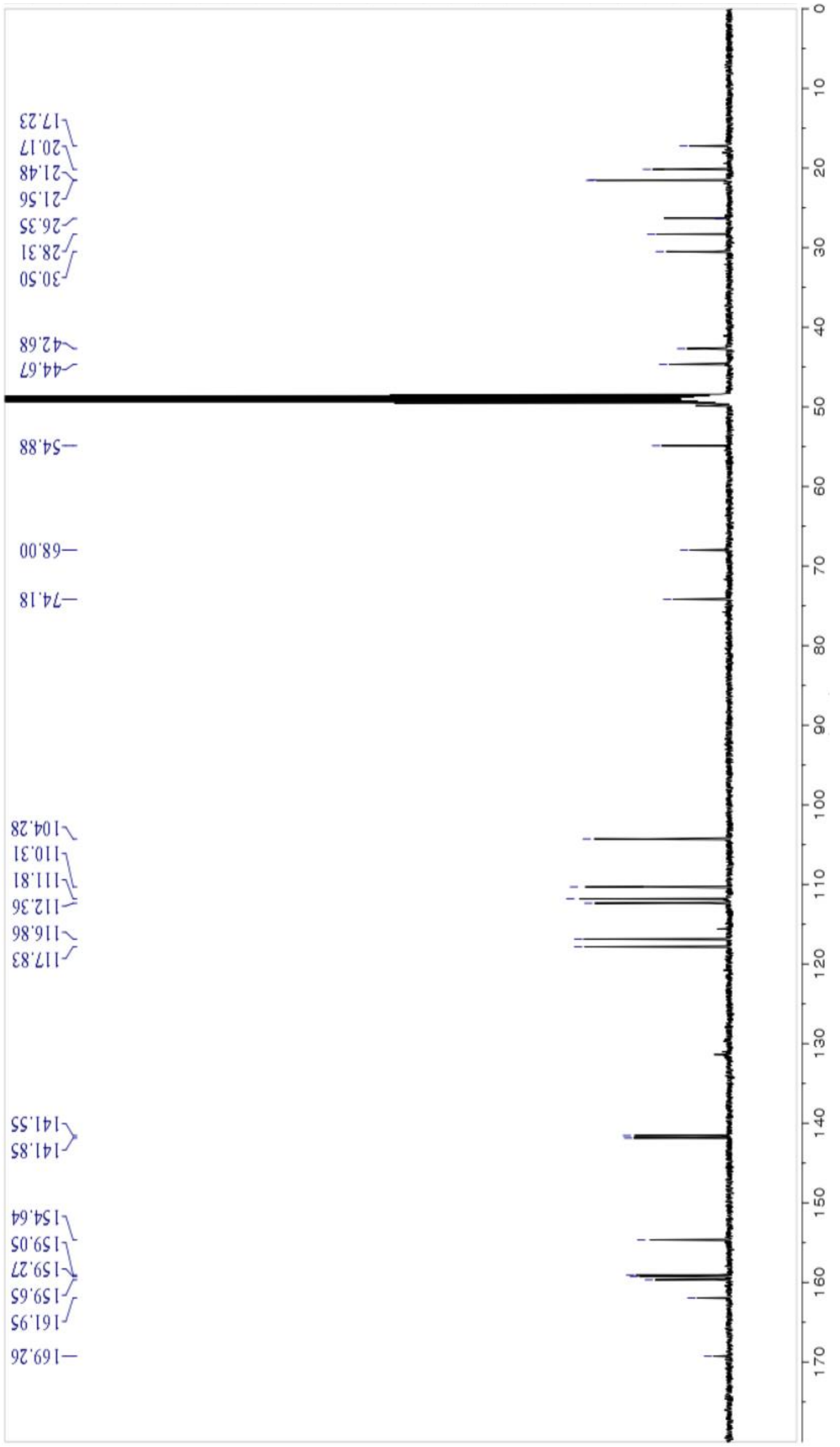

Figure S30. The ^{13}C NMR (125 MHz, MeOH-d_4) spectrum of **1**

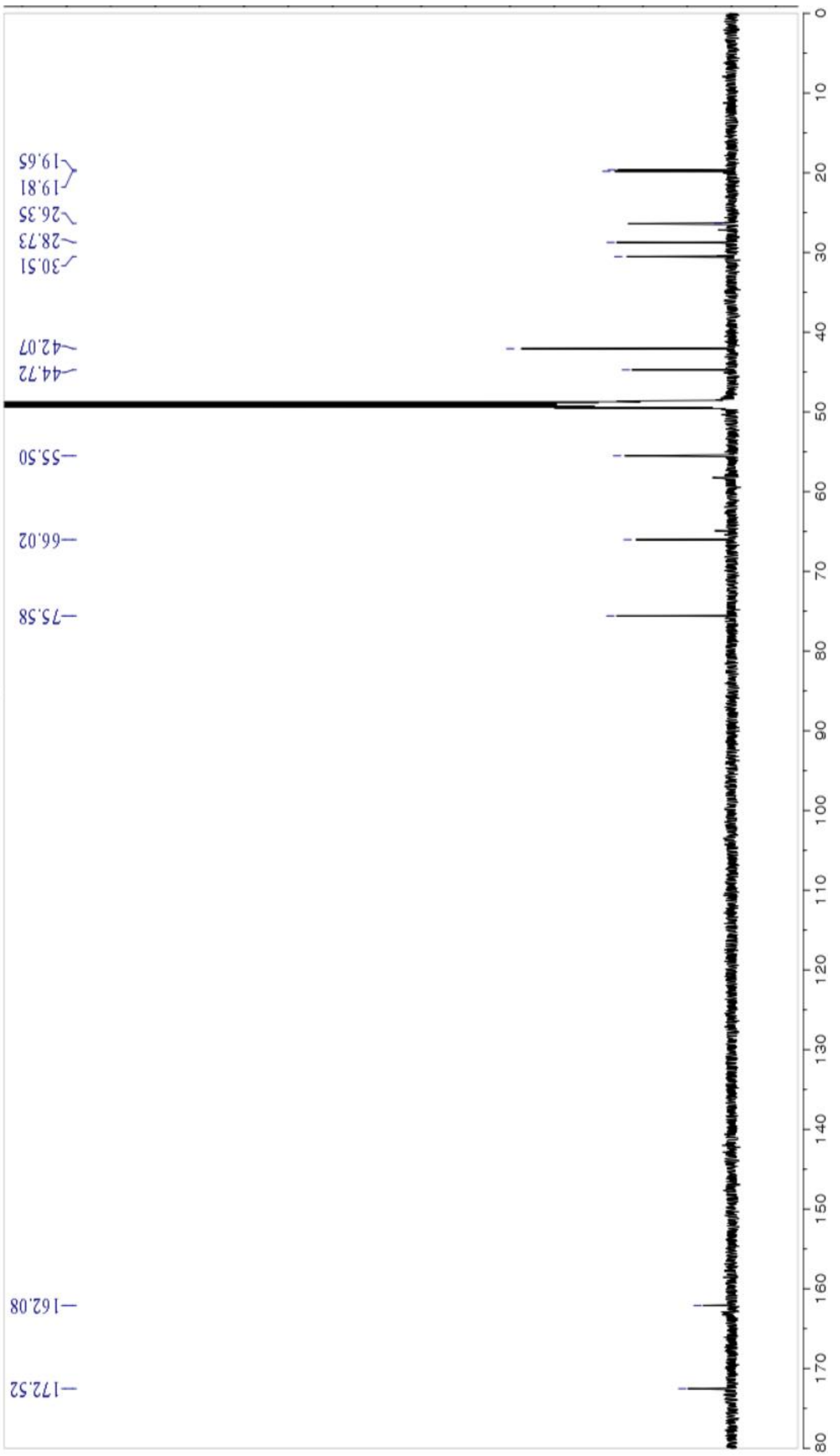

Figure S31. The ^{13}C NMR (200 MHz, MeOH-d_4) spectrum of **3**

Table S2. The ^{13}C NMR data of **1–3** and **3c**

No	1[a]	2[a]	3[a]	3c[a]	1[b]	3[b]
	δ_C, type	δ_C, type	δ_C, type	δ_C, type	δ_C, type	δ_C, type
1	154.50, C	154.50, C			154.64, C	
2	108.13, CH	108.15, CH			110.31, CH	
3	156.65, C	156.69, C			154.64, C	
4	113.93, CH	113.89, CH			116.86, CH	
5	139.28, C	139.34, C			141.55, C	
6	115.83, CH	115.95, CH			117.83, CH	
7	157.59, C	157.60, C			159.05, C	
8	102.74, CH	102.79, CH			104.28, CH	
9	158.43, C	158.47, C			159.27, C	
10	109.82, CH	109.85, CH			111.81, CH	
11	140.05, C	140.10, C			141.85, C	
12	111.15, CH	111.21, CH			112.36, CH	
13	21.02, CH_3	21.13, CH_3			21.48, CH_3	
14	21.09, CH_3	21.06, CH_3			21.56, CH_3	
1′	42.67, CH_2		42.74, CH_2	42.80, CH_2	44.67, CH_2	44.72, CH_2
2′	24.78, CH_2		24.82, CH_2	25.18, CH_2	26.35, CH_2	26.35, CH_2
3′	28.96, CH_2		28.98, CH_2	29.22, CH_2	30.50, CH_2	30.51, CH_2
4′	52.98, CH		52.95, CH	52.88, CH	54.88, CH	55.50, CH
5′	64.45, CH_2		64.64, CH_2	66.11, CH_2	68.00, CH_2	66.02, CH_2
6′	170.53, C		170.37, C	ND	169.26, C	172.52, C
7′	73.31, CH		73.19, CH	72.26, CH	74.18, CH	75.58, CH
8′	26.84, CH		26.80, CH	26.57, CH	28.31, CH	28.73, CH
9′	19.13, CH_3		19.16, CH_3	17.78, CH_3	17.23, CH_3	19.65, CH_3
10′	19.51, CH_3		19.16, CH_3	19.57, CH_3	20.17, CH_3	19.81, CH_3
11′	41.01, CH_3		41.05, CH_3	41.46, CH_3	42.68, CH_3	42.07, CH_3
12′	41.01, CH_3		41.05, CH_3	41.46, CH_3	42.68, CH_3	42.07, CH_3
13′	160.24, C		160.39, C	161.47, C	161.95, C	162.08, C

[a] Data were obtained in DMSO-d_6. [b] Data were obtained in MeOH-d_4.
